# RNA G-quadruplex formation in biologically important transcribed regions: can two-tetrad intramolecular RNA quadruplexes be formed?

**DOI:** 10.1093/nar/gkae927

**Published:** 2024-11-04

**Authors:** Pritha Basu, Iva Kejnovská, Martin Gajarský, Denis Šubert, Tereza Mikešová, Daniel Renčiuk, Lukáš Trantírek, Jean-Louis Mergny, Michaela Vorlíčková

**Affiliations:** Institute of Biophysics of the Czech Academy of Sciences, Královopolská 135, 612 65 Brno, Czech Republic; Institute of Biophysics of the Czech Academy of Sciences, Královopolská 135, 612 65 Brno, Czech Republic; Center for Molecular Medicine Cologne CMMC, University of Cologne, Robert-Koch-Str. 21, 50931 Cologne, Germany; Central European Institute of Technology, Masaryk University, Kamenice 735/5, 625 00 Brno, Czech Republic; Institute of Biophysics of the Czech Academy of Sciences, Královopolská 135, 612 65 Brno, Czech Republic; National Centre for Biomolecular Research, Faculty of Science, Masaryk University, Kamenice 735/5, 625 00 Brno, Czech Republic; Institute of Biophysics of the Czech Academy of Sciences, Královopolská 135, 612 65 Brno, Czech Republic; Department of Biochemistry, Masaryk University, Kamenice 735/5, 625 00 Brno, Czech Republic; Institute of Biophysics of the Czech Academy of Sciences, Královopolská 135, 612 65 Brno, Czech Republic; Central European Institute of Technology, Masaryk University, Kamenice 735/5, 625 00 Brno, Czech Republic; Institute of Biophysics of the Czech Academy of Sciences, Královopolská 135, 612 65 Brno, Czech Republic; Laboratoire d’Optique et Biosciences, Ecole Polytechnique, CNRS, INSERM, Institut Polytechnique de Paris, 91120 Palaiseau, France; Institute of Biophysics of the Czech Academy of Sciences, Královopolská 135, 612 65 Brno, Czech Republic

## Abstract

G-quadruplexes (G4s) formed within RNA are emerging as promising targets for therapeutic intervention in cancer, neurodegenerative disorders and infectious diseases. Sequences containing a succession of short GG blocks, or uneven G-tract lengths unable to form three-tetrad G4s (GG motifs), are overwhelmingly more frequent than canonical motifs involving multiple GGG blocks. We recently showed that DNA is not able to form stable two-tetrad intramolecular *parallel* G4s. Whether RNA GG motifs can form intramolecular G4s under physiological conditions and play regulatory roles remains a burning question. In this study, we performed a systematic analysis and experimental evaluation of a number of biologically important RNA regions involving RNA GG motifs. We show that most of these motifs do not form stable intramolecular G4s but need to dimerize to form stable G4 structures. The strong tendency of RNA GG motif G4s to associate may participate in RNA-based aggregation under conditions of cellular stress.

## Graphical Abstract

**Graphical Abstract:**
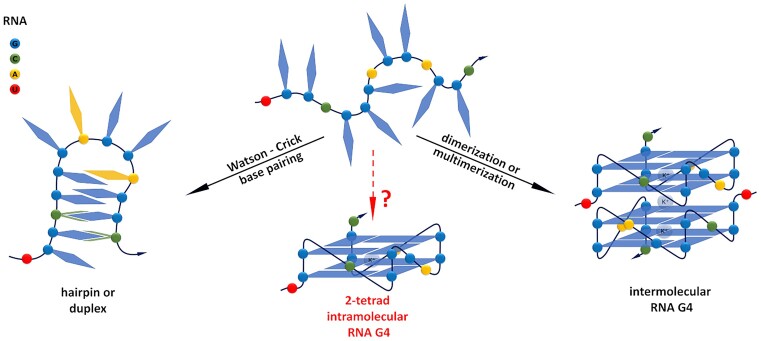

## Introduction

G quadruplexes (G4s) are non-canonical structures formed by Guanine-rich DNA and RNA sequences (1–3). Their structure and stability depend on the sequence and environment. G4s result from the stacking of two or more G-tetrads formed via guanine—guanine Hoogsteen base pairing (4). G-rich sequences can be found in the genomes of most prokaryotes, eukaryotes and viruses, with G4 formation confirmed *in vivo* in several species (5–7). G4s play important roles in various normal and pathological processes, including regulation of gene expression, replication and the maintenance of genome integrity (8). Therefore, G4s constitute important therapeutic targets in many pathologies, including cancer, neurodegenerative disorders or infectious diseases (9–11).

A G-tetrad (or G quartet) is the elementary building block of G4s, which is formed by the association of four guanines held together by an array of eight Hoogsteen-type hydrogen bonds. These tetrads stack over each other to form a quadruplex. The overall thermal stability of G4s largely depends upon the oligonucleotide sequence, the type and concentration of stabilizing metal cations and the number of G tetrads: as the number of G-tetrads increases, G4s usually become more stable (12). Most of the stable G4s involve three or more tetrads, but stable antiparallel intramolecular two-tetrad DNA G4s have also been reported (13–15). G4 stability further depends upon the bases that are not involved in G-tetrad formation and either constitute the loops linking contiguous runs of guanines or belong to flanking nucleotides or bulges (2,16–18) and may participate in terminal capping base pairs or triads.

G4s can be mono-, bi-, tri- or tetra-molecular (19,20). Depending on the relative orientations of the four strands, G4s can adopt parallel, antiparallel or hybrid topologies (21,22) in relation to the loop type. In the case of RNA G4s (rG4s), the 2′-OH groups favour a C3’-*endo* sugar pucker, allowing strong stacking interactions between the tetrads and, therefore, markedly increasing RNA G4 stability (23). This C3’-*endo* sugar pucker favours an *anti*-orientation of the guanine bases, restricting the G4 topology to parallel.

DNA and RNA sequences containing four blocks of Gn, where *n* ≥ 3 interrupted by 1–7 nucleotides (nt), i.e., the ‘classical’ G4 sequence consensus, are known to form stable G4s, generally involving at least three tetrads. G4 formation with shorter GG motifs has been investigated for over two decades (see Supplementary Table S1 for some examples). Yet, whether two tetrad G4s are stable under physiological conditions and play regulatory roles is still under scrutiny (24). This is especially important, given that we anticipated that GG blocks would be overwhelmingly more common than GGG blocks, meaning that there are far more motifs compatible with two-tetrad than with three-tetrad G4s. Sequences containing GG blocks or a succession of G-tracts of uneven length, thus not allowing the formation of ‘regular’ three-tetrad G4s are called ‘GG motifs’ in the following text, or QP2-7 in our bioinformatic studies.

At the DNA level, the study of G4 formation on model sequences G_2_(T_x_G_2_)_3_ (with *x*= 1–7) revealed that the formation of antiparallel two-tetrad G4s was possible for *x*= 2–4 while G4 formation was unfavourable for sequences with longer loops (25). Sequences with a single T in loops were found to form parallel G4s (26–28), but the presence of multiple electrophoretic bands revealed the formation of intermolecular higher-order structures. A recent publication from our group showed that, in contrast to antiparallel G4 structures (such as the stable thrombin aptamer), two-tetrad intramolecular parallel DNA G4s are unstable (29). At the RNA level, structural information on GG motifs is limited, and the principles governing their folding are poorly understood. Theoretical studies indicate that RNA is more prone to form short parallel two-tetrad G4s than DNA (30). Several studies of rG4s with sequences allowing the formation of only two tetrads were undertaken (Supplementary Table S1), but a systematic experimental evaluation of RNA two-tetrad propensity and stability is currently lacking. In particular, molecularity was studied only in a handful of cases. Notably, in the only two cases where nuclear magnetic resonance (NMR) allowed an unambiguous determination of molecularity, rG4s were found to be dimeric (31).

Structural information on these sequences is therefore still limited, and the principles governing their folding are poorly understood. Whether RNA sequences containing GG motifs can form *intramolecular* two-tetrad RNA G4 remains unknown and, regarding their high abundance in biologically important RNA regions, a matter of ongoing debate (24). To assess *intramolecular* two-tetrad G4 formation in RNA sequences containing GG motifs in this issue, we decided to systematically study or reanalyse a variety of RNA GG motifs, some of them having previously been predicted or experimentally tested, including those natively present in the TRF2 and ADAM mRNAs, and SARS-CoV-2 genome (31–33).

Based on systematic studies of these biologically relevant RNA sequences using biophysical and biochemical techniques, we conclude that *intramolecular* two-tetrad RNA G4s are unstable and tend to associate to form stable intermolecular G4 structures. Combining several methods was essential to provide robust conclusions on G4 formation and molecularity. Our findings provide important insights regarding the potential biological roles of RNA GG motifs; presented data suggest that these motifs might constitute the basis of long-range associations within the same RNA to bring distal parts of a single RNA strand together to form a biologically active 3D structure or associations between different RNAs, which are relevant to liquid-liquid phase separation (LLPS) connected with the formation of stress-induced membrane-less organelles and transcription silencing. Our data are important for all those studying the functional consequences of G4 formation in selected RNA regions and will hopefully help prevent the accumulation of erroneous work based on G4 misidentification.

## Material and methods

### Bioinformatics analysis of QP motifs frequency

The number of QP2-7 and QP3-7 motifs in RNA/genomic sequences was analysed using R-package pqsfinder (34). The QP sequences matching the motif G_R_N_L_G_R_N_L_G_R_N_L_G_R_, where G stands for guanines in G-block and N refers to nucleotides in loops (including guanines), were searched as follows: The length of loops L was set in range from 1 to 7. R (length of the G-track) was specified as 2 for QP2-7 and 3 for QP3-7. Only non-overlapping motifs with a minimum score of 20 and no bulges, mismatches or defects were considered. The annotated genomic regions or RNA sequences were analysed on the coding strand only, while the search for QP motifs in human genome was performed on both strands. All other search parameters were set to default values. The frequencies of QP2-7 and QP3-7 motifs were calculated as the average occurrence per Mb of corresponding region. The sequences of mRNAs and viral genomes were obtained from NCBI repository (35). lncRNA sequences were downloaded from RNA central database (36) and human lncRNA database LncBook_v2 (37). The NCBI annotation tracks (38) of 3′UTRs and 5′UTRs specific for human genome assembly GRCh.38, also referred to as hg.38, were obtained using UCSC Genome Browser (39). The sequences of corresponding regions were generated by BedTools (40), from hg.38 assembly. The QP2-7/QP3-7 motif abundancies in human genome were analysed using R-packages BSgenome.Hsapiens.UCSC.hg38 (41) and BSgenome.Hsapiens.NCBI.T2T.CHM13v2.0 (42). CDS and Transcript data are available at the NCBI repository (35). Accession numbers and resources of datasets are summarized in Supplementary Table S2.

### Oligoribonucleotides

The desalted oligoribonucleotides used were purchased from Merck (Haverhill, UK). Some of the RNA sequences (specified) were additionally purified on HPLC. A 1 mM sodium phosphate buffer with 0.3 mM EDTA, pH 7, was used to dissolve the lyophilized oligonucleotides. The precise concentration of the oligonucleotides was determined by measuring the absorption at 260 nm in a 1 mM sodium phosphate buffer with 0.3 mM EDTA at 90°C using molar absorption coefficients calculated as stated in (43). Absorbance was measured on a Specord 250 Plus spectrophotometer (Jena, Germany). The experiments were conducted in 10 mM potassium phosphate buffer supplemented with KCl up to 95 mM (110 mM K^+^ hereafter), pH 7 at 23°C (exceptions are mentioned).

### Circular dichroism (CD)

All the circular dichroism (CD) spectra were recorded in a Jasco 815 spectropolarimeter (Tokyo, Japan). Spectra were measured in the 200–330 nm range with data pitch 0.5 nm and 100 nm/min scan speed with four-scan accumulation. The details of the experimental protocol are given in (29). The measurements were done in a Peltier cell holder at 23°C at two oligonucleotide concentrations: approximately 8 μM (hereafter denoted as ‘*low*’) in 1 cm pathlength Hellma cells and 100 μM or more, when specified (denoted as ‘*high*’ concentration in the rest of the manuscript) in 0.05 cm pathlength rectangular cells. Exact RNA concentrations (related to RNA strands) are given in particular figure legends.

### UV absorption spectroscopy and thermal melting

Melting experiments were performed in a UV/Vis spectrometer Varian Cary 4000 (Mulgrave, Australia) in 1 or 0.05 cm cells in the range of 230–330 nm. The samples were melted 24 h after equilibration upon adding potassium ions. The temperature was increased/decreased by 1°C, and the samples were equilibrated for 2 min at every step before absorption measurement (total time 4.5 min per point, resulting in a ramp rate of 0.22°C/min). The melting curves were basically plotted as the molar absorption ε [M^−1^cm^−1^] at 297 or 260 nm as a function of temperature (44), and the *T*_m_ values were determined as the mid-transition point between native (1) and unstructured (0) states from the normalized melting dependencies. The accuracy of *T*_m_ determination was estimated to be ± 1°C based on repeated measurements.

### Thermal difference spectra (TDS)

Thermal difference spectra (TDS) were calculated as a difference of the absorption spectra corresponding to minimal (ε_MIN_) and maximal (ε_MAX_) molar absorption values at 297 nm (45). Unless stated otherwise, TDS were calculated from the first run of melting.

### Fluorescence spectroscopy

Fluorescence excitation spectra were recorded with a Chirascan Plus fluorimeter (Applied Photophysics, Leatherhead, UK). For studying the interaction of G4 forming sequences with *N*-methylmesoporphyrin IX (NMM), we prepared NMM in the experimental buffer (46). At first, a steady state fluorescence spectrum of 2 μM NMM was measured, and then gradually, RNA samples were added up to 1:1, 1:2, 1:3 and 1:4 concentration ratios to reach an RNA final concentration of 8 μM, and the spectra were measured. Excitation was set to 399 nm, and the emission spectra were recorded in the 400–700 nm wavelength range.

### NMR spectroscopy

Samples were denatured in 1 mM Na-phosphate buffer to remove possible associates formed in stock solution and then transferred (unless stated otherwise) into 25 mM Bis-Tris buffer, pH 6.8 and 110 mM KCl at a strand concentration of about 100 μM. The precise concentration is given in particular figure legends. CD was checked before each NMR measurement. 1D ^1^H NMR spectra were measured at 700 or 950 MHz using a Bruker Avance III HD spectrometer equipped with a triple-resonance cryogenic probe. The NMR spectra were recorded at 20°C upon the addition of 10% D_2_O using a WATERGATE pulse sequence (47). The spectra were processed with TopSpin v4.0.6 (Bruker, USA) and MNova v14.3.3 (Mestrelab Research, Spain).

### Analytical Ultra Centrifugation (AUC)

Samples 15 μM in 10 mM K-phosphate + 35 mM KCl (50 mM K^+^) were prepared in 0.1 cm cells similarly as for CD measurement (in some cases specified in the text Analytical Ultra Centrifugation (AUC) was measured with samples diluted from NMR measurements). Sedimentation velocity was measured on ProteomeLab XL-I (Beckman Coulter) at 20°C (samples in the rotor equilibrated for 1.5 h at this temperature before the measurement). The measurement was undertaken in a double sector centerpiece cell, titanium, with an optical path length of 3 mm (Nanolytics Instruments) at the rotor speed of 60,000 rpm. The detection was set to 200 scans in 5-min intervals, ABS scanning details were 260 nm, continuous mode, radial size increment 0.003 cm. Data were analysed in Sedfit 15.01c, model: continuous c(s) distribution data corrected for ‘time stamp errors’.

### Reverse transcription experiments

Template and primer sequences used for the experiment were obtained from Merck. Lyophilized oligonucleotides were dissolved in DEPC diH_2_O to a 10 mM nucleoside concentration stock solution. The dsDNA was prepared by three-step PCR (3 min at 94°C initial denaturation, 35 cycles: 30 s/94°C denaturation, 30 s/50°C annealing, 30 s/72°C extension, 10 min/72°C final extension and cooling at 4°C) using 100 ng of single-stranded template, 50 μM primers (Ext T7-prim-FWD and Ext T7-prim-REV, final concentration 0.5 μM) and Taq DNA polymerase 1.1 (Top-Bio). PCR products were purified by QIAquick columns (Qiagen) and diluted in 50 μl of elution buffer. RNA templates were prepared using the HiScribe T7 In Vitro Transcription Kit (New England Biolabs) and purified using the Monarch RNA Cleanup Kit (New England Biolabs). DNA reverse primer EXT T7-prim-REV with template AAU GGU3 were labelled by ^32^P using γ-^32^P-ATP and T4 PNK (New England Biolabs). ^32^P-labelled DNA was purified by CentriSpin 10 column (Princeton Separations), and then specific activity was measured.

Reverse transcription by SuperScript IV: RNA templates (5 pmol, final concentration: 0.5 μM) and ^32^P-labelled primer (1 pmol, final concentration 0.1 μM) were used for each reverse transcription reaction. AAU GGU3 was pre-incubated in two different concentrations (0.5 and 5 μM) to encourage an intermolecular G4 formation at higher concentration. The 5 μM RNA was incubated in reaction buffer overnight and then diluted to the final 0.5 μM RNA. The remaining templates were incubated only at a lower concentration for 60 min. Template and primer mixtures were added to three concentrations of NMM (0, 10 and 50 μM) and incubated at 30°C for 60 min. Around 0.4 μl of SuperScript IV reverse transcriptase (ThermoFisher Scientific) was added, and the mixtures were incubated at 30°C for 60 min. The reaction was stopped by the addition of 0.5 μl of 2 M NaOH and thermal denaturation at 95°C for 5 min. The cDNA was purified by ethanol-acetate precipitation and dissolved in 2 μl of loading dye (bromophenol blue, xylene cyanol and formamide) and denatured at 95°C for 5 min.

Denaturing PAGE of reverse transcriptase products and quantification: 1.5 μl of denatured samples were loaded onto pre-heated polyacrylamide gel (16%, 20:1 mono:bis, 7 M Urea, 1 × TBE, 40 cm long). Electrophoresis was performed for 105 min at 45 W in 1 × TBE. The gel was exposed to a Phosphor imager screen for 16 h, and the image was digitalized by a Typhoon FLA 9500 device. The percentages of full products (yellow frame) and G4 pausing sites (red frame) were calculated using ImageQuant TL analysis software, as a ratio of band intensity to the cumulative of the migration line.

## Results

### Bioinformatics analyses

To confirm the prediction that GG motifs are far more frequently found in RNA (as well as in DNA) than sequences containing GGG or longer G blocks, we analyse a number of genes and viral genomes (Table 1) and extended these observations to the complete human genome (Supplementary Table S3).

**Table 1.** Occurrence of GG motifs (QP2-7) and GGG motifs (QP3-7) quadruplexes in representative RNAs using pqsfinder (34) with loops of 1–7 nt and runs of 2 or 3 guanines

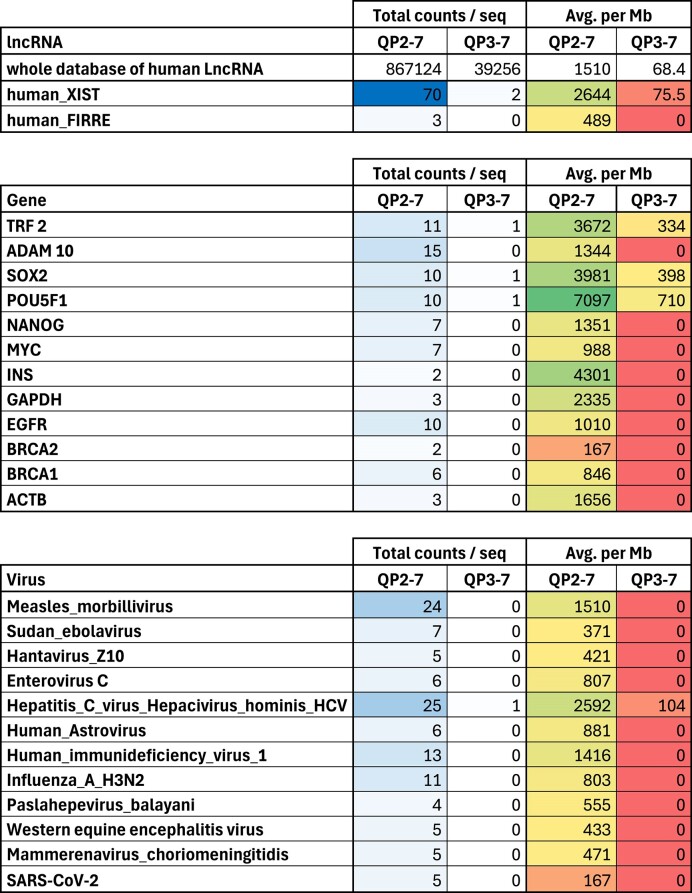

Total counts per sequence are color-coded from none (white) to high abundancy (dark blue). Frequencies are represented from red (none) to green (high density). Data provided for long non-coding RNAs (*top*), human messenger RNAs (*middle*) and various RNA viruses (*bottom*). Analysis of coding strand only, without overlapping motifs. Sequences obtained from corresponding databases (accession numbers are provided in Supplementary Table S2).

The comparison between GG and GGG motifs is exemplified for a number of viral and mRNA sequences in Table 1. Many of these genes or viral genomes do not contain a single sequence allowing for classical three-tetrad G4. In contrast, several GG motifs can be found. At the mRNA level, we found a higher density of GGG and GG motifs in the 5′UTR region, compared to 3′UTR or coding sequence. Within each mRNA region considered, GG motifs were far more abundant (Supplementary Figure S1).

One specific example is provided in Table 2A, which illustrates the motifs found in the 5′UTR region of the TRF2 mRNA. A single canonical motif gggagggcggggaggg (underlined and highlighted in green) has been characterized more than a decade ago (32), while multiple GG motifs also present (highlighted in yellow).

**Table 2.** A. First 400 nt of the TRF2 mRNA containing sequences studied in the paper. B. Oligoribonucleotides used in this study^a^

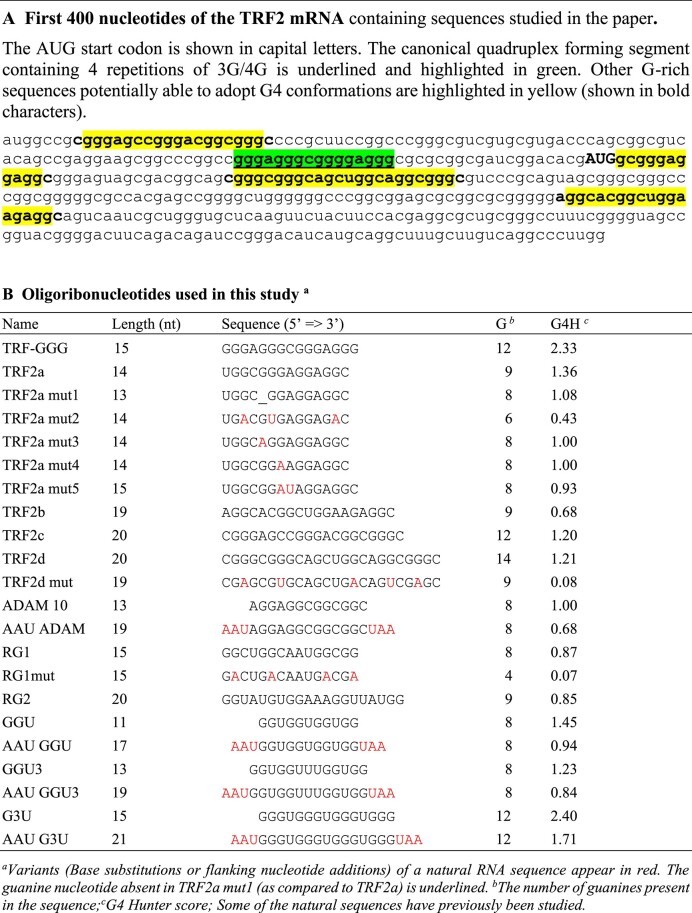

We then proceeded by experimentally testing GG motifs, starting with those found in the TRF2 mRNA.

### The TRF2a RNA sequence and its variants

We started our study with biologically relevant G-rich sequences found in the 5′-UTR region of the TRF2 mRNA (32) (Table 2A). In this region, one can find a single sequence able to form a stable three-tetrad quadruplex (G4) (characterized and compared to DNA G4 in Supplementary Figure S2) and several other G-rich motifs that lack one or more guanines to allow stable three-tetrad quadruplex formation (GG motifs). These sequences represent suitable starting models for our investigation of whether such sequences can adopt stable intramolecular G4s. The motifs selected for the study and their mutated analogues are shown in Table 2B; these include the native sequences found in the 5′-UTR region (TRF2a, TRF2b, TRF2c and TRF2d) and several mutated analogues of TRF2a and TRF2d. Electronic CD, UV absorption, NMR and fluorescence spectroscopies, and AUC were used for their thorough investigation. The results are more extensively discussed with the first sequence studied, TRF2a.

**Figure 1.**
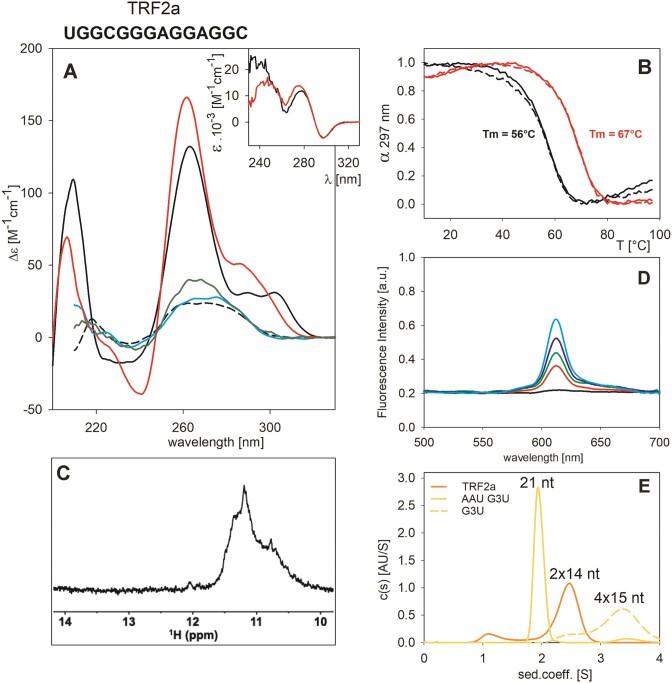
Characteristics of the TRF2a sequence. (**A**) CD spectra at ***low*** RNA concentration (9 μM in this panel) in 110 mM KCl (black), 110 mM LiCl (cyan), 110 mM NaCl (green), 1 mM Na phosphate only (black dashed line), or at ***high*** RNA concentration (190 μM here: lower concentrations provided unresolved NMR spectra of low signal-to-noise ratio-see Figure 2) in 110 mM KCl (red). Insert in (**A**): TDS spectra from melting dependencies measured at (black) *low* and (red) *high* concentrations expressed in molar absorption per strand; (**B**) normalized thermal melting dependencies at 297 nm for (black) *low* and (red) *high* concentrations measured with the same samples as in (**A**). Solid lines and dashed lines correspond to heating and cooling experiments, respectively; (**C**) NMR spectrum of TRF2a measured at 180 μM RNA in 110 mM K^+^; (**D**) fluorescence emission spectra measured at (red to cyan) (2–8) μM RNA : 2 μM NMM; (black) NMM alone. (**E**) AUC measured at 15 μM RNA concentration: TRF2a, a 14-nt-long bimolecular G4 is shown in orange, AAU G3U, a 21-nt long intramolecular G4 is shown as a yellow full line. The position of this G4 is used as a marker for all other AUC experiments. G3U, a tetramolecular G4 formed by four 15-nt long oligoribonucleotides is shown as a yellow dashed line.

**Figure 2.**
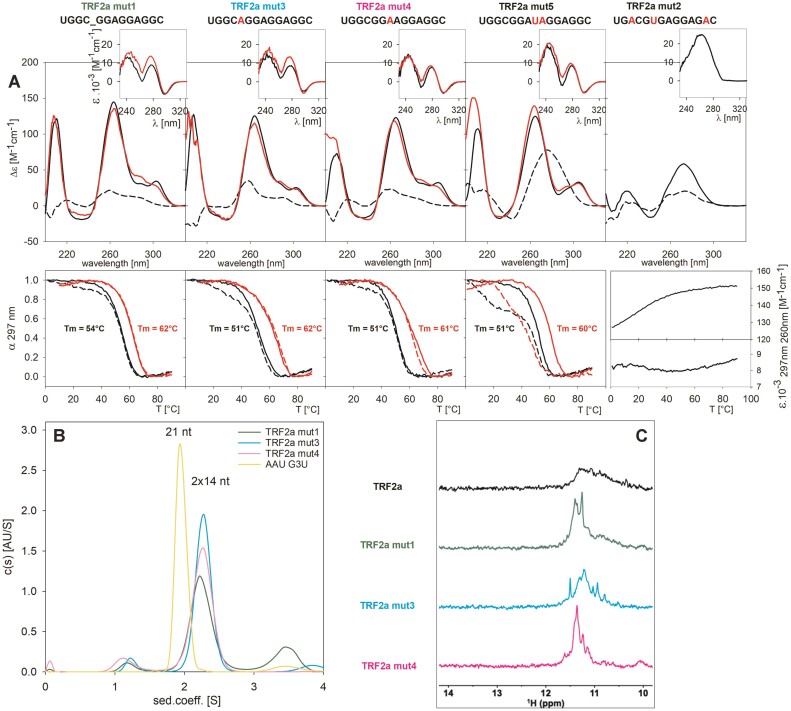
Characteristics of TRF2a mutant sequences. (**A**) The black dashed lines correspond to the CD spectra at *low* (8 μM) RNA concentration. The red lines correspond to *high* (180 μM RNA concentration - same as with TRF2a). CD spectra were recorded in 1 mM Na phosphate and 110 mM K^+^. Inserts in (**A**): TDS spectra. Lower row: temperature dependencies at *low* (black) and *high* (red) concentrations. Solid lines and dashed lines correspond to heating and cooling experiments, respectively; (last panel) molar absorption of mut2 as a function of temperature. (**B**) AUC (15 μM strand concentration) of 13- and 14-nt-long TRF2a mutants as indicated by colours; yellow corresponds to the 21-nt long intramolecular AAU G3U quadruplex standard. (**C**) NMR spectra of 70 μM TRF2a and of its mutated analogues drawn in the colours as denoted in figures.

TRF2a forms a quadruplex in the presence of K^+^ ion as shown by characteristic positive peaks around 265 and 210 nm (Figure 1) (48,49). The CD signal of this sequence in the presence of Na^+^ or Li^+^ is much lower, and it is distinctly different from the G4-specific K^+^ one. (Figure 1A). This confirms that TRF2a forms a quadruplex, but only in the presence of K^+^. In addition to the signal characteristic for G4, the spectrum in K^+^ contains a wide long wavelength positive signal (probably formed by two bands, at ∼285 and ∼300 nm) that is not usually present with standard three-tetrad G4s. This feature has been previously also observed in CD spectra of other two-tetrad RNA G4s (31,50,51). The intensity of the positive 260 nm peak was higher at the highest RNA concentration, which indicates the formation of intermolecular G4s. (The denomination ‘*high*’ and ‘*low*’ corresponds to ∼100 and ∼8 μM, respectively, throughout the paper; higher RNA concentration was used here because of NMR spectrum of low signal-to-noise ratio at 100 μM or lower concentrations; Figure 2). The whole CD spectrum slightly shifted toward blue at *high* concentration, and the shape of the long wavelength region changed with a substantially increased signal at ∼285 nm.

G4 formation is reflected by a characteristic increase in absorbance around 297 nm. Thus, the TDS absorption (45) of TRF2a (Figure 1A, insert) containing negative long wavelength values was in line with the presence of G4. The decrease in absorbance at 297 nm with temperature is a sensitive measure of G4 thermal denaturation (Figure 1B).

We then studied the interaction of TRF2a with N-methyl mesoporphyrin IX (NMM). NMM is a molecule that becomes fluorescent upon binding to G4s, especially to parallel G4s, but not in the case of duplex or single-stranded sequences (52). As shown in Figure 1D, TRF2a induced an increase in fluorescence emission of NMM, suggestive of G4 formation.

The imino region of 1D ^1^H NMR spectrum of TRF2a displayed a broad unresolved signal between 11.5 and 10.5 ppm, which is a region typical for imino hydrogens involved in the Hoogsteen base pairing (Figure 1C). Although the signal position is consistent with G4 formation, the broad and unresolved character of the signal indicates that TRF2a exists in the solution as a mixture of various G4 arrangements, likely including oligomers.

The above-given results allowed us to conclude that TRF2a formed a G4 structure but provided little clue on its molecularity. We thus measured the thermal stability of the TRF2a sequence at different strand concentrations (Figure 1B). At *low* (9 μM) RNA strand concentration, the UV absorption sharply dropped with increasing temperature giving a *T*_m_ value around 56°C, to be compared with a value of 67°C at *high* (190 μM) concentration. The melting profile was fully reversible at high sample concentration, but there was a slight hysteresis in the case of low RNA concentration, probably indicating slower G4 formation. This, along with the higher 260 nm amplitude in the CD spectrum and higher *T*_m_ at high strand concentration, indicates the formation of intermolecular G4s. The sample, however, ran as an intramolecular structure at low RNA concentration on the electrophoresis, and only a minor band was observed corresponding to higher molecular structures (Supplementary Figure S3). We concluded that some of these intermolecular G4s may dissociate during electrophoresis. We observed the same phenomenon with other studied sequences, and thus decided to use analytical ultracentrifugation (AUC) to unambiguously determine molecularity.

To firmly establish molecularity, we performed AUC experiments using three-tetrad intramolecular and intermolecular G4s as controls (Supplementary Figure S4). The (G_3_U)_3_G_3_ (named G3U) sequence forms intermolecular, mainly tetramolecular, G4s. In contrast, the same core sequence, in which trinucleotides were appended at both ends, is less prone to intermolecular associations, as shown previously for other sequences (53,54). This 21-mer AAU(G_3_U)_3_G_3_UAA (named AAU G3U) forms an intramolecular G4 (Supplementary Figure S4). Its NMR spectrum is characteristic of a three-tetrad G4, and its CD spectrum is only slightly lower than that of (G_3_U)_3_G_3_ (Supplementary Figure S4). CD spectra of the two sequences and their positions on the AUC record were compared with those of the classical duplex of (G_3_U)_3_G_3_ RNA with its complementary strand.

Using the AAU G3U 21-mer as an AUC standard and based on the data shown in Figure 1E, we can conclude that the TRF2a sequence forms a G4, mainly as a bimolecular structure. A very minor peak is present at the position corresponding to monomolecular species, which does not necessarily correspond to a G4. Thus, it is unclear whether TRF2a can form a two-tetrad RNA G4 without dimerization.

To find out how slight changes in primary structure may influence G4 folding, we mutated some of the bases in TRF2a (Figure 2): We first mutated the single GGG track in TRF2a (UGGCGGGAGGAGGC) by deleting one guanine and then we substituted the first or the third G in the GGG track by adenine (A) resulting in an elongation of the first or the middle loop, respectively. Finally, we extended the middle loop by inserting a UA dinucleotide, thus resulting in a central AUA loop. All the sequences thus contain only GG (rather than GGG) dinucleotides. CD spectra and TDS confirmed that these sequences formed G4 in the presence of K^+^ ions (Figure 2). The shape of the CD spectra at low strand concentration was similar to that observed with TRF2a. However, while the CD signal at 285 nm of TRF2a increased significantly at high concentration, only a small increase of this band was observed in the case of mut1. An even smaller change was observed for mut4 and the spectrum of the other mutations did not change with RNA concentration. The small dependence of mutated sequences on strand concentration is probably connected with their limited possibilities of forming various G4s, which do not change with concentration.

AUC confirmed (Figure 2B and Supplementary Figure S3) that the G4s were primarily bimolecular. The substitution of the GGG block by GG (mut1) resulted in *T*_m_ decreases by 2 and >5°C at *low* and *high* RNA concentrations, respectively. G4s of the mutated (four GG-containing) sequences mutually differ only slightly; the loop elongation by single nucleotide resulted in a decrease of *T*_m_ by 3°C at *low* and by (1–2)°C at *high* RNA concentration. G4 of the sequence with the extended middle loop (AUA) was formed the least willingly, as reflected by its slow reformation after denaturation (Figure 2). TRF2a mut2 with G for A substitution in every G block, and thus not able to form G4, was used as a control sequence. In line with this, CD and TDS spectra differed distinctly form G4 forming sequences and, in contrast to them, its absorbance at 297 and 260 nm increased with temperature, as observed for denaturation of duplex or other non-G4 structures. No clear thermal transition was provided with mut2. NMR spectra of the mutated sequences revealed better-resolved peaks than TRF2a (Figure 2C), indicating a lower heterogeneity of their G4 structures. Thus, the presence of only 8 guanines in the mutated sequences probably reduced the possibilities of mistaken G interactions and led to more ordered bimolecular folding. This explanation may also hold for the observed relatively small dependence of their CD spectra on concentration.

In contrast to TRF2a, the mutated sequences provide, in addition to bimolecular species, a small signal of tetramolecular structures seen by AUC. Interestingly, a distinct population of the tetramolecular species is formed by mut1, which is 1 nt shorter than the other mutated sequences and, as the only one, contains only single-nucleotide loops.

### Other TRF2 RNA sequences

**Figure 3.**
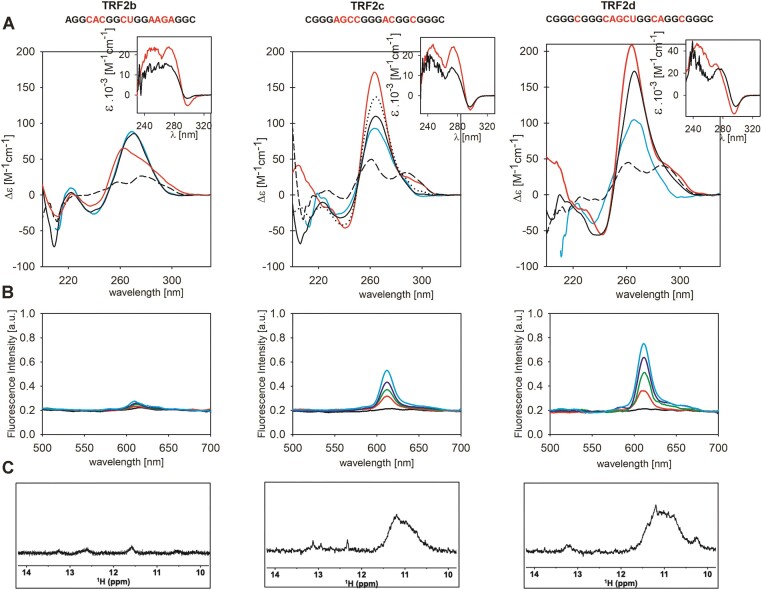
Spectral characteristics of the TRF2b, TRF2c and TRF2d sequences. (**A**) CD spectra at *low* RNA concentrations (8 μM) in (black dash) 1 mM Na phosphate, (cyan) 110 mM Li^+^, (black) 110 mM K^+^ (black dotted) measured after 24 h, and at *high* concentrations (red; 92, 95 and 75 μM for TRF2b, TRF2c and TRF2d, respectively). Inserts: TDS spectra determined at *low* and *high* RNA concentrations; (**B**) fluorescence spectra with variable NMM:RNA ratios from 1:0 to 1:4 (black to cyan). (**C**) NMR spectra for 110 μM RNA were measured in the presence of 110 mM K^+^ immediately after annealing.

Three other natural motifs found in the TRF2 mRNA were then explored: TRF2b, TRF2c and TRF2d (Table 2B, Figure 3). The CD spectra of TRF2b (**left Panel**) exhibited a rather high positive peak around 265 nm and a negative one at 210 nm at low RNA concentration. Such CD spectrum with the negative 210 nm band is indicative of A-form RNA structure (49) rather than G4 formation. The spectra were the same in K^+^ and Li^+^ conditions. A small UV absorption value at 297 nm slightly decreased with increased temperature, while absorption at 260 nm increased (Supplementary Figure S5), contrasting with G4-forming sequences. TDS, however, contained a tiny negative band at 297 nm, indicating a small presence of G4 (Figure 3A, left **insert**), and a very little fluorescence peak appeared upon TRF2b interaction with NMM (**tier B**). An increase in TRF2b concentration led to a decrease of the positive CD band and its shift to 260 nm and, simultaneously, a clear negative 295 nm TDS band appeared and became deeper with time. The G4 of TRF2b is thus formed ‘reluctantly’, better at higher concentrations and with slow kinetics, thus primarily intermolecularly. The imino region of the NMR spectrum of TRF2b shows three broad tiny signals (Figure 3, **tier C**), suggesting that the TRF2b either forms a single structure stabilized by Watson–Crick (signals in region 12–14 ppm) and Hoogsteen (signal at ∼ 11.6 ppm) base pairs or a mixture of different structures, including a small amount of G4. This behaviour may be related to its relatively long loops and the presence of four cytosines in the sequence interfering with G4 formation.

TRF2c (middle **Panel**) displayed an A-like type CD spectrum at a low concentration, similar to that of TRF2b. However, the 260 nm CD band increased after 24 h, and TDS and NMM studies suggested that TRF2c formed a G4. Its CD spectrum at 260 nm, and thus population of G4 increased with time and upon increasing RNA concentration. The observed slow G4 formation and its dependence on RNA concentration reveal the formation of predominantly intermolecular G4s. Similar to the situation with TRF2a, the broad unresolved signals in the imino region of the 1D ^1^H NMR spectrum indicated that TRF2c formed a mixture of different G4 structures. Thermal denaturation of TRF2c (all temperature dependencies were measured the next day after preparation in K^+^) led to a sharp transition. Renaturation was complete upon cooling (temperature gradient 0.22°C/min) but the process displayed a large hysteresis due to a slow kinetics of G4 reformation (Supplementary Figure S5B). Hairpins or duplexes, whose existence is indicated by the presence of distinct signals in the upfield part of the NMR spectrum (12–14 ppm), probably hinder G4 formation. TRF2c contains comparably long loops and more Cs in its primary structure than TRF2b. The reason for its higher susceptibility to form G4 is probably related to more Gs, specifically three GGG runs.

TRF2d (right **Panel**) was still more prone to G4 formation than TRF2c. It adopted a G4 fold even at low strand concentration, according to CD, temperature dependence, TDS and NMM experiments (Figure 3 and Supplementary Figure S5). The broad NMR peaks, however, indicate that the TRF2d quadruplex is also a complex mixture of various G4 structures. G4s are, however, disposed to form relatively more easily, though the sequence contains even more cytosines (but also more guanine) runs than the previous ones.

A mutated variant of TRF2d (Supplementary Figure S5D) was constructed and designated as TRF2d mut so that it should not form a G4 structure. Its CD spectrum contains a high positive band around 265 nm (like TRF2d in Li^+^ and TRF2c in K^+^ at low concentration) and a deep negative band around 210 nm, characteristic of the A-form of RNA (49). UV absorption of the mutated TRF2d increased with temperature, and thus, TDS provided a very high band around 260 nm (and no negative band at 295 nm) (Supplementary Figure S5D), indicating the formation of an ordered, from G4 different, structure. CD and TDS of TRF2d mut differ distinctly from the unstructured form of TRF2 mut2 (Supplementary Figure S6). A sign of cooperativity of its melting curve and its slight two-step course may correspond to the melting of an A-form duplex and a hairpin. Similarly, the A-form structures are formed by TRF2b and by TRF2c immediately after their preparation at low concentration in K^+^. Some duplexes or hairpins remain present even at high concentrations of TRF2d (similar to TRF2c) (Figure 3, **tier C**) in a mixture with G4, as indicated by the small peaks corresponding to W.C base pairs visible on the NMR spectrum in the region between 12 and 14 ppm. Thus, at low strand concentrations, TRF2b, TRF2c and TRF2d adopt hairpins or duplexes, which interfere with G4 formation. TRF2c, with a half number of C than G, forms heterogenous G4 structures with time and especially at high RNA concentration. TRF2d with the same C/G ratio forms G4 even at low concentrations. The G4s formed are surprisingly stable (Supplementary Figure S5), though they appear to have relatively slow kinetics.

Overall, the experiments with TRF2a-TRF2d demonstrate that these RNA may adopt G4 structures. However, the propensity of the individual sequences to adopt a G4 fold depends on the number of Gs susceptible to contribute to the G4 core, the length and primary sequence of nucleotides forming loops, and the capacity of the sequence to form alternative structures such as hairpins, for example. As shown here, the G4 formed are primarily intermolecular; thus, we have not yet found an RNA sequence clearly able to adopt a predominantly *intramolecular* G4 containing only two tetrads. We thus considered other motifs found in biologically relevant RNAs.

### SARS-CoV-2 RG1 and RG2 sequences

**Figure 4.**
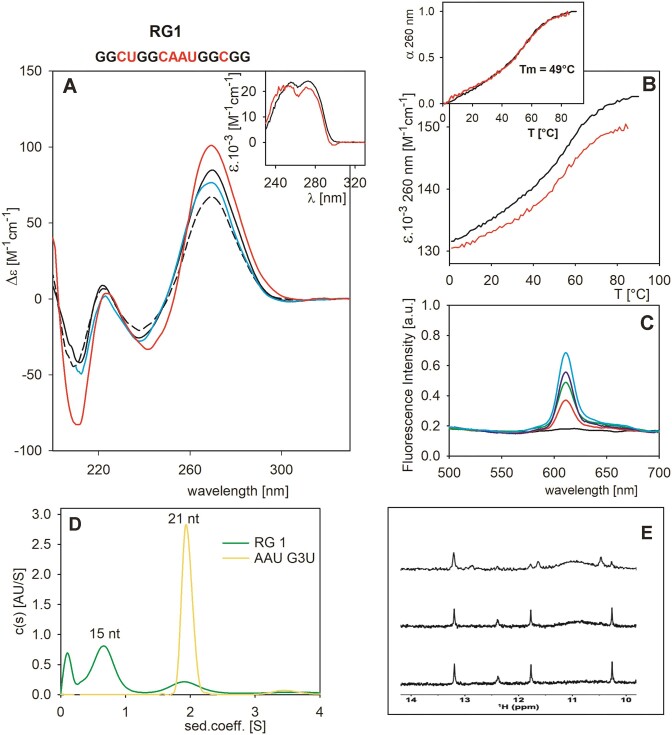
RG1 characterization. (**A**) CD spectra at *low* (8 μM) concentration in 1 mM Na phosphate (black dashed line), 110 mM K^+^ (black), 110 mM Li^+^ (cyan) and at *high* concentration (100 μM) in 110 mM K^+^ (red). Insert in (A): TDS spectra at *low* and *high* concentrations (black and red curves, respectively). (**B**) Temperature dependencies reflected by changes in molar absorbance at *low* (black) and *high* (red) concentrations. Insert in (B): both temperature dependencies run along the same curve after 0–1 normalization (at 0°C and 85°C, respectively). (**C**) Fluorescence spectra: 2 μM NMM with 0–8 μM RNA added (black to cyan). (**D**) AUC spectra of 15-nt long RG1 (15 μM; green) and 21-nt long AAU G3U marker (yellow). (**E**) NMR spectra of 100 μM RG1 in 110 mM K^+^ measured at room temperature (top to bottom) immediately, after 9 days, and after 5 min denaturation at 90°C and quenching on ice.

Two highly relevant RNA GG motifs involving only GG blocks are the RG1 and RG2 sequences, which were reported to form G4 (33,55). CD spectrum of RG1 (Figure 4) exhibits a positive maximum at 260 nm, similar to a quadruplex structure (56,57) but a negative band at 210 nm, in contrast to G4 (49). The spectrum is nearly independent of cation type (K^+^, Li^+^, and even in 1 mM Na phosphate) and does not change with time. This spectrum is also similar to one of its mutated analogues, RG1 mut, in which all four GG are substituted by GA, preventing G4 formation (Supplementary Figure S7). The positive long-wavelength band slightly increased with increasing RG1 concentration, in parallel with a deepening of the negative 210 nm band (Figure 4A). Neither TDS (Figure 4A, insert) nor temperature dependence (Figure 4B) of RG1 (absorbance at 297 and particularly at 260 nm increased with increasing temperature) indicate a presence of G4. These results indicate that RG1 does not form G4 at low RNA concentration, contrasting with previous claims.

Interestingly, the addition of NMM led to a fluorescence increase, in agreement with G4 formation (Figure 4C). AUC reveals (Figure 4D) that RG1 predominantly forms an intramolecular structure and that only a minor amount of the sequence folds intermolecularly. To characterize this intramolecular species, we recorded its NMR and CD spectra and AUC profiles (the AUC measurement was made using the NMR sample immediately after dilution). The imino region of the ^1^H NMR spectra (Figure 4E) acquired immediately after mixing oligonucleotide into the buffer shows two sets of signals; a set of sharp signals dispersed between 10.2 and 13.2 ppm, likely corresponding to a hairpin-like structure and broad unresolved signal centred at ∼11 ppm. The overall appearance of the NMR spectrum indicates that RG1 forms a mix of structures; the broad signal at ∼11 ppm suggests that the mix also includes a low population of various Hoogsteen base pairs stabilized G4 structures. However, the broad NMR signal centred at 11 ppm diminishes with time (Figure 4E), suggesting that G4 species either unfold or convert into hairpins, which may contain three G.C pairs. The temperature dependence of the absorbance at 260 nm (and to a lesser extent at 297 nm) increases even at high RG1 concentration. The course of the dependence shows a hint of cooperativity with the same T*_m_* value for both *low* and *high* RNA concentrations. The predominant intramolecular structure (according to AUC) at ∼0.8 S probably corresponds to a hairpin, while the structure at ∼ 1.9 S may correspond to a minor bimolecular G4 species.

The G4 structure, which we could not detect by CD or absorption, potentially exists as a metastable state that can be stabilized by NMM. As expected, the mutated RG1 analogue, RG1 mut, unable to form G4, exhibited essentially the same CD and TDS spectra with a similar temperature dependence but with no change in fluorescence upon NMM addition, therefore, completely excluding G4 formation (Supplementary Figure S7).

Overall, and contrary to our expectations, analysis of the RG1 sequence did not allow us to conclude about the existence of intramolecular, two-tetrad RNA G4s.

**Figure 5.**
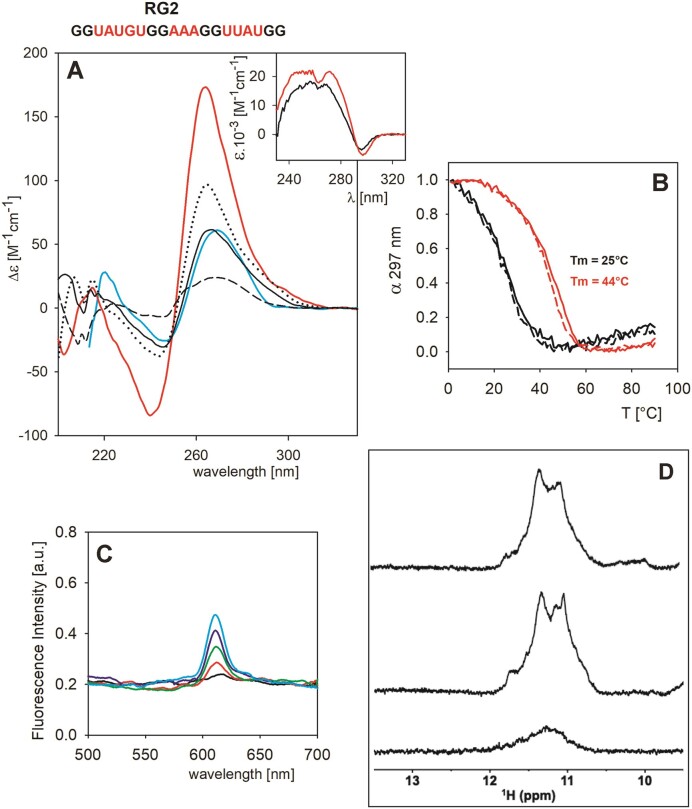
Spectral characteristics of RG2. (**A**) CD spectra at *low* RNA concentration (8 μM) in 1 mM Na phosphate (black dash), in 110 mM K^+^ (black), in 110 mM K^+^ after 24 h and measured at 1°C (black dots) and in 110 mM Li^+^ (cyan) and at *high* concentration (90 μM) in 110 mM K^+^ (red). Insert in (**A**): TDS spectra at *low* (black) and *high* (red) concentrations. (**B**) Temperature dependencies at *low* (black) and *high* (red) concentrations. Solid lines and dashed lines correspond to heating and cooling experiments, respectively. (**C**) Fluorescence spectra of 2 μM NMM with 0–8 μM RNA (black to cyan). (**D**) NMR spectra of RG2 (220 μM concentration) in 110 mM K^+^ measured either immediately (top) or the next day (middle). Bottom spectrum was measured at 97 μM RG2 concentration.

The sequence RG2 contains four GG steps as RG1. Its relatively long loops may hinder G4 formation, but RG2 does not contain cytosines potentially interfering with G4 formation. RG2 forms a G4 structure, as demonstrated here by CD, TDS, and NMR spectra and interaction with NMM (Figure 5). The initial CD spectra at low RNA concentration exhibited relatively low positive CD signal at 260 nm in K^+^ (as compared with the hairpins described above). This spectrum is again like the spectra measured in Li^+^ or in only 1 mM Na phosphate, suggesting that this sequence does not form any organized structure at low concentrations. However, this CD signal increased with time and with decreased temperature, suggesting that the RG2 quadruplex was slowly formed. TDS and thermal melting measured one day after RG2 preparation in 110 mM K^+^ indicates the presence of G4. A stable RG2 G4 was formed upon increasing RG2 concentration. This indicates that the G4 is again formed above all intermolecularly, in line with the strong dependence of its stability on oligonucleotide concentration (Figure 5B). The NMR spectrum measured immediately after preparation at standard ∼100 μM concentration was completely unresolved (Figure 5D, bottom). We thus further increased RG2 concentration by a factor of ≈2 to obtain a better spectrum. The spectrum exhibited a broad and still unresolved signal centred at ∼11.2 ppm (Figure 5D), which slightly improved after 24 h. The slow kinetics of G4 formation and its dependence on RNA concentration indicate formation of G4-based intermolecular structures.

Altogether, analysis of the RG1 and RG2 sequences confirmed unambiguous G4 formation for only one of them (RG2) and suggested a predominantly intermolecular association. Again, no proof for forming an intramolecular two-tetrad G4 could be obtained. We, therefore, turned our attention to a different RNA motif.

### ADAM 10 sequences

**Figure 6.**
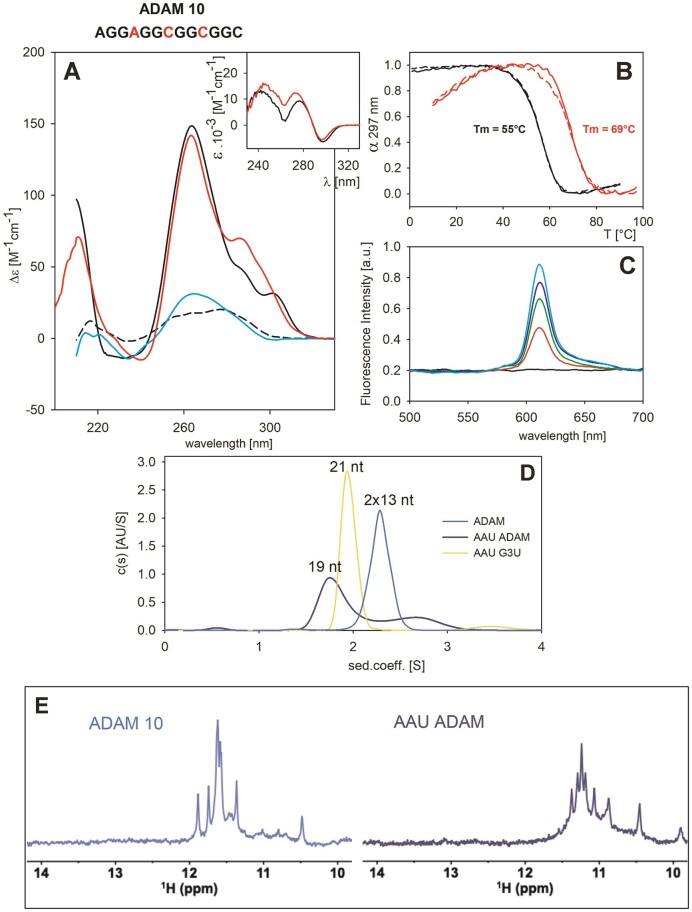
Characterization of the ADAM 10 sequence. (**A**) CD spectra at *low* RNA concentration (8 μM) in 1 mM Na phosphate (black dash), 110 mM KCl (black), 110 mM LiCl (cyan) and at *high* concentration (140 μM) in 110 mM KCl (red). Insert in (**A**): TDS taken from melting dependencies measured at both concentrations expressed in molar absorption per strand. (**B**) Thermal melting dependencies at 297 nm at *low* (black) and *high* (red) concentrations measured with the same samples as in (**A**). Solid lines and dashed lines correspond to heating and cooling experiments, respectively. (**C**) Fluorescence spectra of 2 μM NMM alone (black) or with 2–8 μM RNA (red to cyan), corresponding to 1:1 to 1:4 concentration ratio. (**D**) AUC samples at 15 μM concentration: (blue) two molecules of the 13-nt long ADAM 10, (dark blue) 19-nt long AAU ADAM and (yellow) 21-nt long AAU G3U marker; (**E**) NMR spectra of ADAM 10 and AAU ADAM (100 μM) in 110 mM K ^+^.

ADAM 10 is another previously investigated G4 sequence located in the 5′UTR region of the corresponding mRNA (31). Under conditions identical to those used with previous motifs, ADAM 10 forms a G4 structure as shown by its characteristic CD and TDS spectra (Figure 6A). The G4 is formed even at low concentration, and the height of its 260 nm CD band and TDS spectrum are nearly the same at high RNA concentration. However, the CD signal at 285 nm as well as the stability of this G4 increased substantially with the increase in RNA concentration (Figure 6A, B). NMM fluorescence (Figure 6C) also supports G4 formation. In contrast to the ^1^H NMR spectra of the previously investigated constructs displaying broad and unresolved signals, the ^1^H 1D NMR spectrum exhibited several sharp resolved peaks (Figure 6E) in the region typical for Hoogsteen base pairs imino protons, indicating the formation of a single dominant (stable) G4 structure, while small intensity signals distinctly visible in the region between 10.5 and 11.0 ppm indicate the presence of minor G4 species. The spectrum of the dominant G4 species seems to contain eight imino peaks, the number consistent with the formation of the two-tetrad G-quadruplex or highly symmetric dimeric two-tetrad-based G4 structures. Indeed, the AUC revealed that ADAM 10 forms a bimolecular complex (Figure 6D), which interpretation is in line with the 14°C difference between *low* and *high* concentrations *T*_m_ values and with the literature (31). These results confirm that RNA sequences containing short GG runs adopt intermolecular G4 structures.

Is a two-tetrad RNA G4 able to fold intramolecularly with little or no dimerization? To strongly favour intramolecular folding, we designed an ADAM 10 variant called AAU ADAM, in which trinucleotide flanking sequences (AAU and UAA) were added at both ends, as for our AUC standard, AAU G3U.

AAU ADAM forms a G4 as expected: the shape of its CD resembles one of the bimolecular ADAM 10 (Supplementary Figure S8), but its thermostability under the same conditions is lower than the parent sequence. Its *T*_m_ also depends on RNA concentration with comparable differences between *T*_m_s at *low* and *high* concentrations (48 and 64°C for AAU ADAM, compared to 55 and 69°C for ADAM 10) (Figure 6 and Supplementary Figure S8). The long wavelength CD signal also increased upon increasing AAU ADAM concentration (Supplementary Figure S8) but to a lower extent (and in a wide wavelength interval including 285 and 300 nm bands) than with ADAM 10.

**Figure 7.**
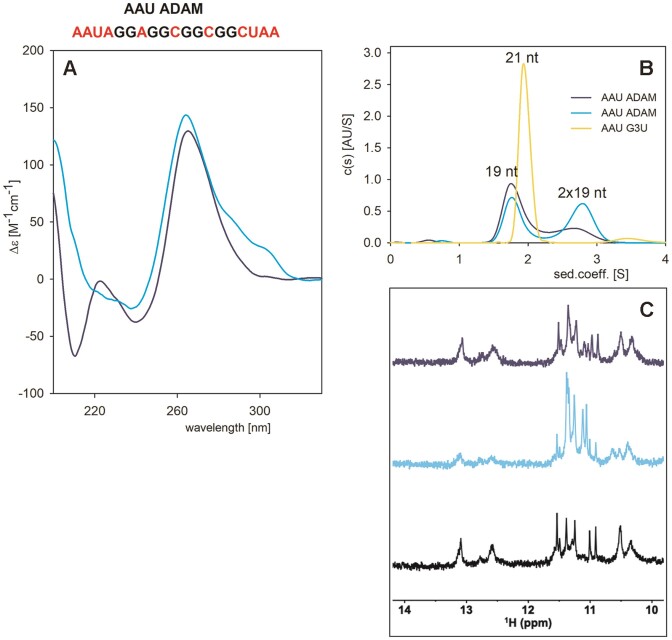
Structural behaviour of AAU ADAM (64 μM strand concentration) in 50 mM K^+^. (**A**) CD spectra; (**B**) AUC: the AAU G3U marker is shown in yellow. (**C**) ^1^H NMR measurements taken immediately after sample preparation (dark blue) or the next day (cyan). For the black curve, the sample was denatured for 5 min at 95°C and the NMR spectrum was taken after a 30 min recovery from denaturation. AUC samples were prepared by diluting the NMR samples to reach a final concentration of 15 μM.

The NMR spectrum of AAU ADAM is also reminiscent of ADAM 10 spectrum (Figure 6E); both spectra show similar patterns and an identical number of peaks (eight) for the dominant species, suggesting the presence of similar two-tetrad-based G4. On the other hand, the AUC signals revealed that AAU ADAM adopts both intra- and inter-molecular structures (Figure 6D) and that the intramolecular structure prevailed with the freshly prepared sample. Interestingly, the AUC peak corresponding to the monomer decreased with time (till the next day), while the bimolecular population increased (Figure 7).

Noteworthy, ADAM 10 and AAU ADAM both contain three cytosines, potentially allowing the formation of stable G.C base pairs, leading to hairpin formation, which may account for the intramolecular species found for AAU ADAM on AUC. We, therefore tried to find out whether the intramolecular species also includes G4. To support intramolecular folding, we used a low-concentration sample of AAU ADAM purified on HPLC and measured simultaneously with the same sample CD, NMR and AUC (Figure 7): The immediately prepared low-concentration sample provided a CD spectrum with a positive band height ∼130 M^−1^cm^−1^ at 260 nm, i.e., comparable to the only desalted sample under the same conditions (Supplementary Figure S8). The CD spectrum contained a clear negative band at 210 nm characteristic of the A-form of RNA, which is not present in the case of pure G4 formed at *high* concentration (Supplementary Figure S8). The corresponding NMR spectrum (Figure 7) measured on a 950 MHz spectrometer showed, in addition to G4-characteristic signals in the 10.2–11.8 region, downfield signals between ∼12.5–13.5 ppm, indicating the presence of structures stabilized by Watson–Crick base-pairs, e.g. hairpins or duplexes. The AUC of this sample provided two bands; the band corresponding to the intramolecular species was much more populated than that corresponding to dimers. Upon re-measurement of the sample after 1 day, the CD spectrum grew slightly at 260 nm, and a positive CD signal appeared on its long-wavelength side. In parallel, the negative 210 nm band disappeared and instead became positive. Simultaneously, AUC revealed an increase in bimolecular species, and NMR evidenced a concomitant increase in G-quadruplex populations. CD, AUC and NMR data indicate that AAU ADAM probably forms a (intramolecular) hairpin-like structure, which converts into G4 dimers with time. After denaturation of the sample by heating at 95°C for 5 min, followed by 30 min lasting cooling to room temperature, the G4 population decreased again and that of hairpins increased. The NMR spectrum of the sample measured immediately essentially recapitulated the spectrum of the freshly prepared sample. G4 formation needs longer time. Altogether, these data suggest that whereas ADAM 10 predominantly forms a bimolecular G4, AAU ADAM forms a mixture of bimolecular G4-based and intramolecular hairpin-based species; the former appears thermodynamically more stable even at low concentrations, but its formation is slow.

### G4 formation with simple (G_2_U)_3_G_2_ and G_2_UG_2_U_3_G_2_UG_2_ model sequences

While the data provided thus far support G-quadruplex formation for GG motif sequences, they do not constitute unambiguous proof of the existence of *intramolecular* two-tetrad RNA G4 structures, as intermolecular G4s prevailed.

To tackle this issue, we studied two additional sequences, (G_2_U)_3_G_2_ and G_2_UG_2_U_3_G_2_UG_2_ (denominated GGU and GGU3, respectively), which allow the formation of only two-tetrad G4s and do not contain cytosines to avoid the formation of competing hairpin or duplex structures.

These two sequences were not included in the previous reports on RNA G4 formation by GG motifs (Supplementary Table S1). We thus first surveyed the frequency of their DNA analogues in the transcribed regions of the human genome to get an idea about their occurrence in the transcriptome. We found that both sequences are significantly more abundant than expected based on the randomized primary sequence and around twice more abundant in transcribed than in non-transcribed regions (Supplementary Figure S9). Detailed analysis of particular gene regions revealed their enrichment in exons, introns and 3′-UTRs, and non-transcribed promoter regions, defined herein as 1000-bp upstream of transcription start site. We observed only a low frequency of both motifs in 5′-UTR (Supplementary Figure S10). The location of GGU and GGU3 within the genome may be, as reported for their three-tetrad analogues (58,59), connected with their biological role.

**Figure 8.**
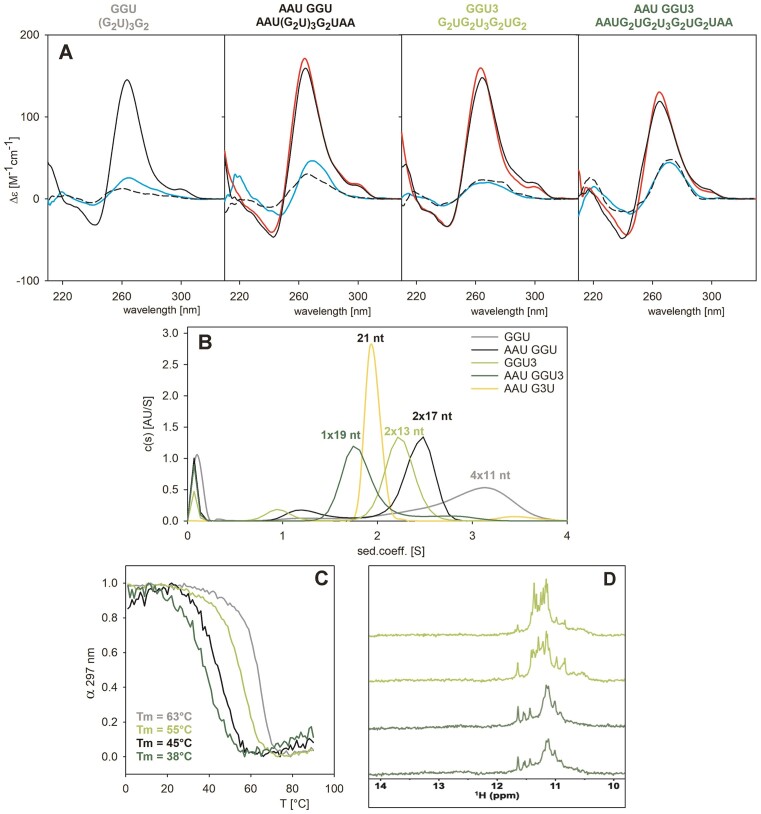
Spectral characteristics of the two-tetrad sequences: GGU (grey), AAU GGU (black), GGU3 (light green) and AAU GGU3 (green). (**A**) CD spectra at *low* (8 μM) RNA concentration in 1 mM Na phosphate (black dash), 110 mM KCl (black), 110 mM LiCl (cyan) or at *high* concentration (140 μM for AAU GGU and 100 μM for the other sequences) in 110 mM KCl (red). (**B**) AUC of the RNA samples (15 μM). The AAU G3U marker is shown in yellow. (**C**) Thermal melting dependencies at 297 nm at *low* RNA concentration. (**D**) NMR spectra of GGU3 and AAU GGU3 (100 μM) measured immediately (upper spectra; the first and the third one) or after one day of incubation (lower spectra; the second and the fourth one).

As shown in Figure 8, the CD spectra of GGU and GGU3, as well as the spectra of their extended analogues (with AAU and UAA added to their 5′ and 3′ends, respectively), are very similar and characteristic of G4 structures. Their positive band at 260 nm is relatively narrow and the long wavelength CD signal is less pronounced than with the previous G4 forming sequences TRF2a or ADAM 10.

The AUC results reveal (Figure 8B) that GGU forms a tetra-molecular structure, while the analog sequence with trinucleotides at both ends forms bimolecular assemblies. Interestingly, elongation of the middle loop led to a decrease in a structure molecularity (Figure 8): while GGU3 forms a bimolecular G4, its extended analog with trinucleotides at both ends is predominantly intramolecular (Figure 8B). Only a very slight population of higher-molecular G4s is visible on AUC. The longer central loop probably confers greater flexibility on the sequence and allows the AAU GGU3 sequence to fold intramolecularly (see also Supplementary Figure S11). The reduction in the molecularity due to the presence of appended sequences is accompanied by a marked and similar decrease in thermostability of both sequences (Figure 8C), by 18°C with GGU and 17°C with the analogue GGU3.

The sequence with U_3_ in the middle loop is thus our best candidate so far for intramolecular two-tetrad RNA G4 formation. Even then, the AUC of GGU3 revealed that the sequence folds into a bimolecular structure. The excessive number of peaks (>10) in the corresponding NMR spectrum (Figure 8D) suggests that GGU3 forms more than one G4-based species at *high* concentration (100 μM) (Figure 8D). Notably, formation of more than one G4-based species is also apparent from the NMR spectrum of the analog with appended trinucleotides (Figure 8D).

**Figure 9.**
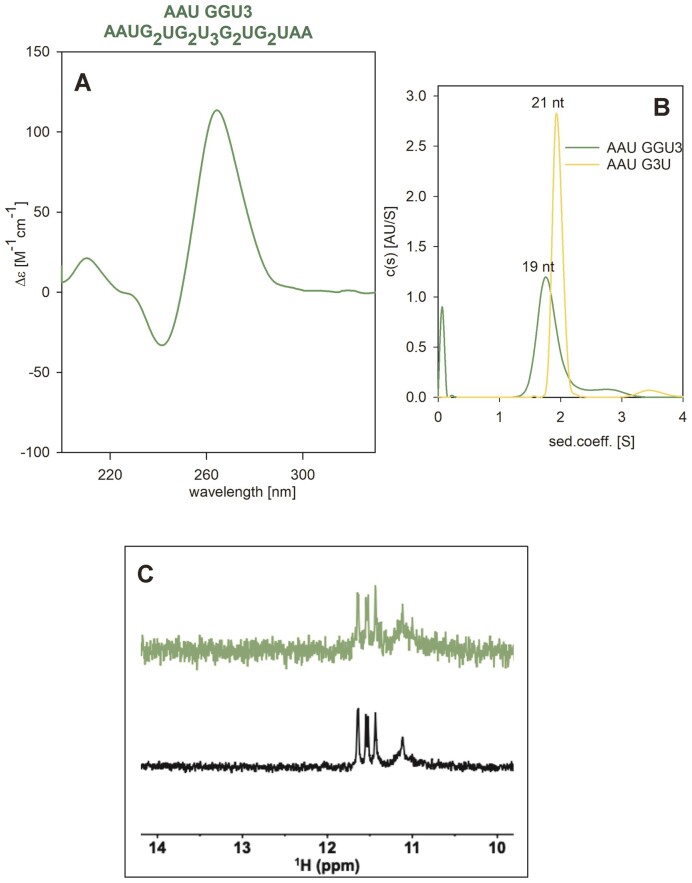
G4 formation of AAU GGU3. (**A**) CD spectra at 10 μM RNA concentration in 110 mM K^+^ (green) immediately before AUC. (**B**) AUC of the same sample (green) and of the AAU G3U marker (yellow). (**C**) NMR spectra of AAU GGU3 measured immediately (green) or upon increasing its concentration to 30 μM, denaturation and 15 min annealing (black).

To support intramolecular folding, we performed the same experiment at a lower concentration, allowing simultaneous measurement of AAU GGU3 on CD, NMR, and AUC (Figure 9). According to these three methods, the sequence unambiguously forms an intramolecular two-tetrad G4: As can be seen in the supplementary Supplementary Figure S12, the structure observed at higher oligonucleotide concentration (Figure 8D) vanishes with the oligonucleotide dilution and with time. As a result, sharp signals around 11.4–11.7 ppm can be seen in Figure 9C corresponding to intramolecular two-tetrad G4. No signals corresponding to other than imino protons of Hoogsteen pairs are present.

Thus, we were finally able to identify one sequence forming an intramolecular two-tetrad quadruplex, at least at low strand concentration. Interestingly, no long wavelength signal is present on its CD spectrum. It is worth noting that the ability of RNA to form parallel intramolecular two-tetrad G4 is not shared by DNA: The analogous DNA sequence G_2_TG_2_T_3_G_2_TG_2_ forms a bimolecular G4, while its extended sequence AATG_2_TG_2_T_3_G_2_TG_2_TAA remains intramolecular and does not form any G4 at all: no signal was observed in the Hoogsteen ^1^H 1D NMR region (29).

## Discussion

Several studies of G4s with sequences allowing the formation of only two tetrads have been published; yet no systematic experimental evaluation of RNA two-tetrad propensity and stability was performed.

### GG-motifs are abundant in the transcriptome

As shown in Tables 1 and S3, GG motifs, which do not allow the formation of ‘regular’ three-tetrad G4s, are far more frequently found in RNA than sequences containing GGG or longer G blocks. This was exemplified in a number of viral and mRNA sequences in which not a single sequence allowing for classical three-tetrad G4 was found, while several GG motifs are present. Whether these motifs actually allow RNA G4 formation i*n vitro* or in cells remains a matter of debate. For example, the G4RP-seq in cell rG4-profilling method, which involves G4 precipitation by the G4-specific probe BioTasQ, identified rG4 sites both in TRF2 and ADAM 10 genes in the MCF7 human cell line (60,61). It is, however, unclear whether the probe specifically recognized the two-tetrad rG4s, as three-tetrad rG4 motifs are also present. Another transcriptome-wide rG4-profilling method, rG4-seq, based on reverse transcriptase stalling *in vitro*, identified over 13,000 rG4 sites in HeLa cells transcriptome (62). Among these, 5222 were two-tetrad rG4, including three sites in the ADAM 10 gene but none in the TRF2 mRNA, despite the presence of evolutionary-conserved three-tetrad motif forming a very stable quadruplex, shown to inhibit translation (32). In any case, rG4-profiling confirmed G4 formation for a few thousands of GG-motifs, demonstrating the relevance of these sequences.

### GG-runs are reluctant to form intramolecular quadruplexes

Using a combination of biophysical and biochemical tools such as analytical ultracentrifugation, CD and NMR spectroscopies, we showed that RNA sequences deficient in guanines and incapable of forming stable three- or four-tetrad intramolecular G-quadruplexes (GG motifs) generally tend to associate through dimerization (and hypothetically multimerization), even at low micromolar concentrations. The strong tendency of the GG motifs to multimerize probably relates to our present demonstration that these sequences are not able to fold into stable intramolecular G4s. In line with this observation is the fact that no two-tetrad G4 monomer was found among high-resolution RNA quadruplex structures available in the Nucleic Acids Knowledge Base (NAKB https://www.nakb.org). Some exceptions correspond to super-complex architectures (long aptamers) involving multiple additional interactions, thus not really ‘two-tetrad only‘ RNA G4s.

In contrast, seven structures were found to be dimers of two-tetrad quadruplexes (Supplementary Table S4), meaning that four-tetrad bimolecular assemblies were actually observed. The general tendency of the unstable two-tetrad RNA G4s is, therefore, to dimerize or multimerize. This is an important observation, as such motifs are frequent in the transcriptome, and intermolecular binding may favour long-range associations between different RNAs.

The fact that RNA G-rich sequences containing some GG steps (instead of all GGG or longer G tracts) (GG motifs) do not form stable G4s within a single RNA strand is fundamentally important to anyone interested in the functional consequences of G4 formation. Intramolecular quadruplex formation in the G-rich regions in the K^+^ solutions is generally considered as a certainty, although this assumption may lead to fundamentally flawed conclusions. An aggravating factor is that G4 formation, if checked *in vitro*, relies on CD spectroscopy, in which a CD spectrum with a high positive peak at 260 nm is considered as a proof. However, any A-form RNA duplex displays a similar CD spectrum with a predominant positive band at the same wavelength, and RNA hairpins are thus often misinterpreted as intramolecular G4s. We demonstrate in this work the differences between these otherwise very similar spectra. Indeed, intramolecular two-tetrad RNA quadruplex has not really been demonstrated in articles published so far (Table 1 and Supplementary Tables S3 and S4). Our unequivocal findings may help preventing a flurry of flawed studies on the functional consequences of quadruplex formation.

In addition, we revealed a new feature in the CD spectra of the two-tetrad G4s: the presence of a wide long-wavelength positive CD signal (including bands around 285 and 300 nm), which is not usually observed with standard three-tetrad G4s. Its presence coincides with unresolved NMR spectra of a bulk of natural sequences and is thus probably related to the heterogeneity of their quadruplex arrangement. This heterogeneity may include various irregularities in the formation of G tetrads, G.C pairs, free guanines, distorted and single stranded regions. Of note, antiparallel quadruplexes including antiparallel RNA (63) as well as denatured/single stranded forms of DNA and RNA show a positive signal at 290 nm. The described shape of the CD spectrum of two-tetrad G4s was previously observed (31,50,51), but the presence of the positive long wavelength signal was not commented. We believe that apart from irregularities in G4 structures (rather reflected at 280 nm), the long-wavelength positive CD band (especially at 300 nm) possibly reflects the way single G4s are stacked into dimers.

Despite the technical hurdles and the strong and rather general tendency of RNA GG motifs to oligomerize, we eventually found an intramolecular two-tetrad G4, using a specially designed RNA sequence, which is in agreement with previous theoretical studies (30). Even then, this sequence ultimately dimerizes over time. We cannot decide whether all intermolecular G4s of the studied GG motifs arise from the dimerization of unstable two-tetrad G4s or whether they arise directly from the interaction of more molecules.

### Quadruplex ligands may stabilize these quadruplexes

Of note, G4 probes such as NMM are known to stabilize G4 formation (52) and may actually induce G4 formation if conformationally allowed. This observation illustrates the limitation of using G4-binding probes (e.g. antibodies, small compounds or peptide domains) as evidence for G4 formation since they may displace the equilibrium towards the G4 species.

In our case, NMM stabilizes G4 even when little or no G4 was formed in its absence: metastable forms may be present though, and these G4 species (of unknown molecularity) may be stabilized by NMM to become detectable. Other ligands or G4-binding proteins, including antibodies, can behave in the same way and drive G4 formation.

While this is an experimental problem when attempting to demonstrate G4 formation, this also represents for the cell a potential way to modulate gene expression based on ligand (protein)-induced RNA G4 formation. This also opens new perspectives for natural regulation, as the presence of G4-specific proteins able to recognize these metastable structures may induce G4 formation.

### Interference of GG sequences with biological processes

Our results presented so far indicate only low incidence of intramolecular two-tetrad G4, usually close to or below the detection limit of the methods used, but the equilibrium might be shifted towards the G4 formation by an addition of a G4-stabilizing ligand, NMM. We thus tested the ability of selected G4s to inhibit a progression of SuperScript IV reverse transcriptase, so called RT-stalling experiment (62), both in the absence and presence of NMM. The potential occurrence of intermolecular species was further reduced by lowering the concentration of DNA templates (1 μM), compared to the spectroscopic experiments, and by adding 10 nt on both sides corresponding to the native genomic sequence. These sequences, however, contain additional guanines or even short G-tracts that might interfere with or stabilize the tested G4.

We compared Reverse transcriptase stalling by a three-tetrad AAU G3U, which serves as a positive control, TRF2a and its mutant, RG1 and its mutant and AAU GGU3 at two concentrations. We show (Supplementary Figure S13) that the two-tetrad TRF2a G4 delays transcription less efficiently than the three-tetrad one but more than the mutant unable to form a G4. RG1, which, according to our results, does not form G4 at low concentration, behaves as its mutant whose sequence does not allow G4 formation. AAU GGU3, which forms an intramolecular G4 at low concentration, has no effect on RT stalling but its stalling potential increases in the presence of NMM. In the presence NMM it hinders RNA polymerase action in a similar way as its G4 prepared at ten times higher concentration which stabilizes intermolecular G4 folding.

### Biological roles and formation in cells of RNA G-quadruplexes

The biological roles of RNA G-quadruplexes depend on their location within the transcript, as recently shown for alternative splicing (58), or as enhancers of bacterial translation (59). Note, however, that little is known regarding the specific role of two-tetrad rG4s, which are the focus of this manuscript. Studies performed on three-tetrad rG4s suggest their involvement in key biological processes, as their abundancy within the functional regions of mRNA is significantly higher than elsewhere. Functional studies on mammalian cell models have shown that presence of rG4s in the 3′ or 5′ UTRs have either a silencing or enhancing effect on post-transcriptional gene expression regulation (64–66) rG4s also take part in alternative splicing, as shown recently (58). These roles have been supported by interaction analysis of rG4s, where proteins of splicing apparatus and ribosomal subunits were mostly identified as rG4-selective binding partners (67). Similarly, rG4s have been found to act at the 5′UTRs of bacteria (59). Regarding the function of two-tetrad rG4s, initial works points to their function at the 3′UTR of the unique immediate early gene of Pseudorabies virus (PRV) (68). Among two-tetrad rG4s, while GGU and GGU3 motifs have not yet be assigned a biological role, rG4-seq analysis (62) allowed the identification of no less than 74 GGU motifs (25 in 3′ UTR; 6 in 5′ UTR; 40 and 2 in coding and non-coding sequences, respectively, 1 overlapping 5′ UTR/CDS). The GGU3 motif was identified only once in the 3′ UTR of the RAD18 gene. These results confirm that GGU and GGU3 motifs may fold into G-quadruplex structures *in vitro*.

### Biological implications of multimerization

Another possible consequence of the intrinsic low stability of two-tetrad RNA intramolecular G4s is their strong tendency to oligomerize, which may be relevant for LLP separation associated with the formation of stress-induced membrane-less organelles and transcription silencing. This proposition underscores the potential significance of RNA-RNA interactions and intermolecular G-quadruplex structures in stress granule formation. It aligns with observations of purified cellular RNA forming *in vitro* assemblies analogous to stress granules (69) and findings from the Ivanov group indicating that only sufficiently long expansion of hexanucleotide GGGGCC repeats from the first intron of C9ORF72 can induce phase transitions (granule assembly) both *in vitro* and in cells (70). A role for GG motif-based intermolecular quadruplexes in LLP separation was recently proposed, and recent observations demonstrated the negative regulation of stress granule assembly through unwinding RNA G-quadruplex structures by BLM (71) and DHX36 (72) helicases. This suggests that the intermolecular G-quadruplexes based on GG motifs may serve as substrates for these helicases and contribute to RNA transcriptional silencing under conditions of cellular stress (72).

## Conclusions

To study the functional consequences of G4 formation, it is essential to establish whether an expected quadruplex is actually formed and understand its properties. By studying several naturally occurring motifs found in human mRNA, we evaluated G4 formation of sequences lacking some guanines for building three guanine tetrads. We found that most sequences studied here either did not form any quadruplex or formed bimolecular G4s. Our previous calculations, however, suggested that RNA could be able to form two-tetrad G4 within a single molecule.

Finding one such intramolecular G4 structure was far more difficult than anticipated, and experimental data had to be carefully interpreted to unambiguously demonstrate its formation, as both circular dichroism spectra and fluorescent light-up effects with G4 probes may be incorrectly interpreted. For example, NMM stabilizes G4 and displaces the equilibrium towards it, even in cases when the respective sequences would hardly form any G4 while for CD, an RNA A-form gives spectra that can often be misinterpreted as a G4.

We eventually found one RNA sequence unambiguously forming an intramolecular two-tetrad G4 structure. This G4, however, also dimerized over time to form a stable intermolecular structure. In addition, long loops and the presence of cytosines in the studied sequences favour alternative folds involving Watson–Crick pairing. These observations have profound implications when searching for ‘low stability’ RNA G4s. Intermolecular structures imply the association of several RNA regions, providing the basis for long-range interactions or aggregation.

## Supplementary Material

gkae927_Supplemental_File

## Data Availability

The data underlying this article are available in the article and in its online supplementary material.
