# Supplementary material for: RNA G-quadruplex formation in biologically important transcribed regions: can two-tetrad intramolecular RNA quadruplexes be formed?
Source: Nucleic Acids Res. 2024 Nov 4;52(21):13224–42. doi: 10.1093/nar/gkae927 (PMC11602125; doi:10.1093/nar/gkae927)
Supplement: gkae927_Supplemental_File [file gkae927_supplemental_file.docx]

**RNA G-Quadruplex Formation in Biologically Important Transcribed Regions: Can Two-Tetrad Intramolecular RNA Quadruplexes be formed?**

Revised Sup. Information – Aug. 30, 2024

Pritha Basu^1&^, Iva Kejnovská^1&^, Martin Gajarský ^2,3^, Denis Šubert^1,4^, Tereza Mikešová^1,5^, Daniel Renčiuk^1^, Lukáš Trantírek^3^, Jean-Louis Mergny^1,6^* and Michaela Vorlíčková^1^*

*^1^ Institute of Biophysics of the Czech Academy of Sciences, Královopolská 135, 612 65 Brno, Czech Republic.*

*^2^* *Center for Molecular Medicine Cologne CMMC, University of Cologne, Robert-Koch-Str. 21, 50931 Cologne, Germany.*

*^3^ Central European Institute of Technology, Masaryk University, Kamenice 735/5, 625 00 Brno, Czech Republic.*

*^4^ National Centre for Biomolecular Research, Faculty of Science, Masaryk University, Kamenice 735/5, 625 00 Brno, Czech Republic.*

*^5^ Department of Biochemistry, Masaryk University, Kamenice 735/5, 625 00, Brno, Czech Republic*

*^6^ Laboratoire d’Optique et Biosciences, Ecole Polytechnique, CNRS, INSERM, Institut Polytechnique de Paris, 91120 Palaiseau cedex, France.*

^&^ Equal contribution.

** Authors to whom correspondence may be addressed:*

*E-mail: mifi@ibp.cz;* [*jean-louis.mergny@polytechnique.edu*](mailto:jean-louis.mergny@polytechnique.edu)

**Supplementary information**

**Supplementary Tables S1-S4**

**Supplementary Figures S1-S13**

**Supplementary Table S1**: Previous reports on RNA G4 formation by GG motifs

| Sequences ^[[1]](#footnote-1)^ | Methods ^[[2]](#footnote-2)^ | Comments | Ref. |
| --- | --- | --- | --- |
| R14 **GG**A**GG**UUUU**GG**A**GG** | CD, NMR | Dimerization via Hexad-Hexad interface | [1] |
| G2U **GG**U**GG**AA**GG**A**GG**U**GG**UUCAU**GGG**  G2U2 **GG**UU**GG**AA**GG**A**GG**UU**GG**UCAU**GGG** | CD, CD melting, Gene expression | Tm not concentration-dependent => likely intramolecular | [2] |
| GQP2 **GG**U**GG**U**GG**U**GG**  TBA-RNA **GG**UU**GG**UGU**GG**UU**GG** | CD, CD melting | concentration dependency not tested | [3] |
| Wt **GG**A**GG**A**GGGGG**A**GG**A**GG**A | CD, DMS + RNAse T1 footprinting, luciferase | No information on molecularity | [4] |
| G2L111 **GG**A**GG**A**GG**A**GG**A G2L444 **GG**AGCC**GG**AGUC**GG**AAU**GGG** | Bioinformatics, CD, UV melting, PAGE |  | [5] |
| G2A A**GG**A**GG**A**GG**A**GG**A  G2AA **GG**AA**GG**AA**GG**AA**GG**  G2UA **GG**UA**GG**UA**GG**UA**GG**  G2AUA **GG**AUA**GG**AUA**GG**AUA**GG**  G2L444 **GG**AGCC**GG**AGUC**GG**AAU**GGG** | CD K^+^ folding, PAGE, RNAse T1 |  | [6] |
| FBS_Q1 **GG**A**GGGGG**A**GG**AA**G**A FBS_Q2 **GG**ACAA**GG**A**GG**AAGA**GG**AC | NMR, PAGE, UVmelt | independent of RNA conc. | [7] |
| VEGF **GG**A**GG**A**GGGGG**A**GG**A**GG** | RT Stop |  | [8] |
| I-8 **GG**U**GG**U**GG**AAU**GG** | CD, PAGE | More likely to be intermolecular based on PAGE | [9] |
| OAZ2-5Q2 **GG**C**GG**U**GG**C**GG**CC**GGGG**A**GG**UCAGUU**GG**  AZIN1-5Q **GG**ACCCAGACAUA**GG**CUU**GG**U**GG** | QGRS, UV melting, CD, NMR | some with hysteresis, others not. | [10] |
| SHR **GG**UUU**GG**AGC**GG**U**GGG** | CD, K^+^ dependency, RT stalling | No information on molecularity | [11] |
| ADAM10 A**GG**A**GG**C**GG**C**GG**C  EP300 C**GG**C**GG**C**GG**C**GG**  UEP300U UC**GG**C**GG**C**GG**C**GG**U | NMR, CD, CD melting | Propensity to Aggregate, equilibrium with hairpin. ADAM 10 is a **dimer** | [12] |
| AT4G30480 **GG**A**GG**A**GG**AUAU**GG**  Os02g **GG**AA**GG**A**GG**A**GG**AGCA**GG** | rG4-Seq |  | [13] |
| hemL-wt **GG**TCC**GG**TCTATCA**GG**C**GGG**T  Bsw-R CT**GG**CCAT**GG**TCCTCCA**GG**TCCCCAT**GG**CC | rG4-Seq, ThT, CD | rG4-Seq mostly picks G2 motifs | [14] |
| 11 candidates, relatively long, mostly G2 | Bioinformatics, Inline probing, NMM fluor |  | [15] |
| miR432 UCUU**GG**AGUA**GG**UCAUU**GGG**U**GG**  Kras GC**GG**C**GG**C**GG**A**GG**CA  SC1 GU**GG**AA**GG**AGU**GG**CU**GGG**  SC3 AA**GG**UA**GGG**U**GG**UU**GG**  SC5 GA**GG**AGUU**GG**AA**GG**AU**GGG** | CD, UV-melt, PAGE | Authors conclude intramolecular, but evidence not very strong: Tm somehow conc. Independent, single band on PAGE but hard to conclude – no size markers | [16] |
| RG1 **GG**CU**GG**CAAU**GG**C**GG**  RG2 **GG**UAUGU**GG**AAA**GG**UUAU**GG** | NMM, ThT fluo, FRET NMM, CD, NMR |  | [17] |
| RNA.G4.1 U**GG**CU**GG**CAAU**GG**C**GG**U  RNA.G4.2 U**GG**A**GG**A**GG**UGUUGCA**GG**A | Bioinformatics, NMR, CD | No information on molecularity | [18] |
| Wt U**GG**UACAAU**GGG**U**GG**AAAAU**GGGG**U  Rad54 A**GG**A**GG**A**GG**A**GG**A | rG4-Seq, CD | No information on molecularity | [19] |
| RG1 **GG**CU**GG**CAAU**GG**C**GG** | smFRET, CD | smFRET in agreement with intramolecular, but the system disfavors intermolecular assembly (and performed at very low concentration) | [20] |
| OR4 **GG**A**GG**UUUU**GG**A**GG** | UV melting, CD | Tm = 59.9°C | [21] |
| **GG**C**GG**A**GG**C**GG**AA**GG**AU**GG**C**GG**A**GG**  **GG**U**GG**AU**GG**C**GG**AA**GG**A**GG**C**GG**A**GG**AC**GGG**  **GG**U**GG**UU**GG**A**GG**AC**GG**AU**GG**AC**GG**A**GG**UUUAUUA**GG**  **GG**CU**GG**A**GG**U**GG**AC**GG**AU**GG**CU**GG**A**GG**CGAAAUU**GG** | SELEX | Mostly (GGN_1-3_)_8+_ repeats;  Seq allows double G4 formation | [22] |
| miR-638 A**GGG**AUCGC**GGG**C**GGG**U**GG**C**GG**CCU | UV-melt, NMM, ThT, PAGE | Structure likely to be intramolecular, but sequence should allow formation of 3-tetrads | [23] |

References to Table S1

1. Liu, H., et al., *A dimeric RNA quadruplex architecture comprised of two G:G(:A):G:G(:A) hexads, G:G:G:G tetrads and UUUU loops.* Journal of Molecular Biology, 2002. **322**(5): p. 955-970.

2. Wieland, M. and J.S. Hartig, *RNA quadruplex-based modulation of gene expression.* Chemistry & Biology, 2007. **14**(7): p. 757-763.

3. Joachimi, A., A. Benz, and J.S. Hartig, *A comparison of DNA and RNA quadruplex structures and stabilities.* Bioorganic & Medicinal Chemistry, 2009. **17**(19): p. 6811-6815.

4. Morris, M.J., et al., *An RNA G-quadruplex is essential for cap-independent translation initiation in human VEGF IRES.* Journal of the American Chemical Society, 2010. **132**(50): p. 17831-17839.

5. Mullen, M.A., et al., *RNA G-Quadruplexes in the model plant species Arabidopsis thaliana: prevalence and possible functional roles.* Nucleic Acids Research, 2010. **38**(22): p. 8149-8163.

6. Mullen, M.A., S.M. Assmann, and P.C. Bevilacqua, *Toward a digital gene response: RNA G-quadruplexes with fewer quartets fold with higher cooperativity.* Journal of the American Chemical Society, 2012. **134**(2): p. 812-815.

7. Blice-Baum, A.C. and M.R. Mihailescu, *Biophysical characterization of G-quadruplex forming FMR1 mRNA and of its interactions with different fragile X mental retardation protein isoforms.* RNA, 2014. **20**(1): p. 103-14.

8. Cammas, A., et al., *Stabilization of the G-quadruplex at the VEGF IRES represses cap-independent translation.* RNA Biology, 2015. **12**(3): p. 320-329.

9. Huang, H., et al., *RNA G-quadruplex secondary structure promotes alternative splicing via the RNA-binding protein hnRNPF.* Genes Dev, 2017. **31**(22): p. 2296-2309.

10. Lightfoot, H.L., et al., *Control of the polyamine biosynthesis pathway by G2-quadruplexes.* eLife, 2018. **7**: p. e36362.

11. Zhang, Y., et al., *G-quadruplex structures trigger RNA phase separation.* Nucleic Acids Research, 2019. **47**(22): p. 11746-11754.

12. Binas, O., I. Bessi, and H. Schwalbe, *Structure validation of G-rich RNAs in noncoding regions of the human genome.* Chembiochem, 2020. **21**(11): p. 1656-1663.

13. Yang, X., et al., *RNA G-quadruplex structures exist and function in vivo in plants.* Genome Biology, 2020. **21**(1): p. 226.

14. Do, N.Q., et al., *G-quadruplex structure of an anti-proliferative DNA sequence.* Nucleic Acids Research, 2017. **45**(12): p. 7487-7493.

15. Turcotte, M.-A., et al., *Guanine Nucleotide-Binding Protein-Like 1 (GNL1) binds RNA G-quadruplex structures in genes associated with Parkinson’s disease.* RNA Biology, 2021. **18**(9): p. 1339-1353.

16. Mou, X. and C.K. Kwok, *Effect of RNA sequence context and stereochemistry on G-quadruplex-RHAU53 interaction.* Biochemical and Biophysical Research Communications, 2020. **533**(4): p. 1135-1141.

17. Zhao, C., et al., *Targeting RNA G-Quadruplex in SARS-CoV-2: A promising therapeutic target for COVID-19?* Angew Chem Int Ed Engl, 2021. **60**(1): p. 432-438.

18. Belmonte-Reche, E., et al., *Potential G-quadruplexes and i-Motifs in the SARS-CoV-2.* PLoS One, 2021. **16**(6): p. e0250654.

19. Dumetz, F., et al., *G-quadruplex RNA motifs influence gene expression in the malaria parasite Plasmodium falciparum.* Nucleic Acids Res., 2021. **49**(21): p. 12486-12501.

20. Mukherjee, S.K., J.M. Knop, and R. Winter, *Modulation of the conformational space of SARS-CoV-2 RNA quadruplex RG-1 by cellular components and the amyloidogenic peptides α-Synuclein and hIAPP.* Chemistry, 2022. **28**(9): p. e202104182.

21. Kotkowiak, W., C. Roxo, and A. Pasternak, *Physicochemical and antiproliferative characteristics of RNA and DNA sequence-related G-quadruplexes.* ACS Medicinal Chemistry Letters, 2023. **14**(1): p. 35-40.

22. Zutterling, C., et al., *The forkhead DNA-binding domain binds specific G2-rich RNA sequences.* Nucleic Acids Res., 2023. **51**(22): p. 12367-12380.

23. Lyu, K. and C.K. Kwok, *A G-quadruplex structure in microRNA interferes with messenger RNA recognition and controls gene expression.* Chemical Communications, 2023. **59**(53): p. 8230-8233.

**Supplementary Table S2:**

Sources and accession numbers of datasets used for bioinformatics analysis

| **ID** | **lncRNA** |  |
| --- | --- | --- |
| [lncRNA_human_LncBookv2_OnlyLnc](https://ngdc.cncb.ac.cn/lncbook/downloads) | whole database of human LncRNA |  |
| [URS000025784F_9606](https://rnacentral.org/rna/URS000025784F/9606) | human_XIST |  |
| [URS0000D5A858_9606](https://rnacentral.org/rna/URS0000D5A858/9606) | human_FIRRE |  |
|  |  |  |
| **ID of transcript** | **Gene** | **Transcript variant** |
| [NM_005652.5](https://www.ncbi.nlm.nih.gov/nuccore/NM_005652) | TRF 2 | - |
| [NM_001110.4](https://www.ncbi.nlm.nih.gov/nuccore/NM_001110.4) | ADAM 10 | 1 |
| [NM_003106.4](https://www.ncbi.nlm.nih.gov/nuccore/NM_003106.4) | SOX2 | - |
| [NM_002701.6](https://www.ncbi.nlm.nih.gov/nuccore/NM_002701.6) | POU5F1 | 1 |
| [NM_024865.4](https://www.ncbi.nlm.nih.gov/nuccore/NM_024865.4) | NANOG | 1 |
| [NM_002467.6](https://www.ncbi.nlm.nih.gov/nuccore/NM_002467.6) | MYC | 1 |
| [NM_000207.3](https://www.ncbi.nlm.nih.gov/nuccore/NM_000207.3) | INS | 1 |
| [NM_002046.7](https://www.ncbi.nlm.nih.gov/nuccore/NM_002046.7) | GAPDH | 1 |
| [NM_005228.5](https://www.ncbi.nlm.nih.gov/nuccore/NM_005228.5) | EGFR | 1 |
| [NM_000059.4](https://www.ncbi.nlm.nih.gov/nuccore/1813836564) | BRCA2 | 1 |
| [NM_007294.4](https://www.ncbi.nlm.nih.gov/nuccore/NM_007294.4) | BRCA1 | 1 |
| [NM_001101.5](https://www.ncbi.nlm.nih.gov/nuccore/NM_001101.5) | ACTB | - |
|  |  |  |
| **ID** | **Virus** | **Genome** |
| [GCF_000854845.1](https://www.ncbi.nlm.nih.gov/datasets/taxonomy/11234/) | Measles_morbillivirus | ssRNA(-) |
| [GCF_000855585.1](https://www.ncbi.nlm.nih.gov/datasets/taxonomy/186540/) | Sudan_ebolavirus | ssRNA(-) |
| [GCF_000855785.1](https://www.ncbi.nlm.nih.gov/datasets/taxonomy/93830/) | Hantavirus_Z10 | ssRNA(-) |
| [GCF_000861165.1](https://www.ncbi.nlm.nih.gov/datasets/taxonomy/138950/) | Enterovirus C | ssRNA(+) |
| [GCF_000861845.1](https://www.ncbi.nlm.nih.gov/datasets/taxonomy/3052230/) | Hepatitis_C_virus_Hepacivirus_hominis_HCV | ssRNA(+) |
| [GCF_000861865.1](https://www.ncbi.nlm.nih.gov/datasets/taxonomy/1868658/) | Human_Astrovirus | ssRNA(+) |
| [GCF_000864765.1](https://www.ncbi.nlm.nih.gov/datasets/taxonomy/11676/) | Human_immunideficiency_virus_1 | ssRNA-RT |
| [GCF_000865085.1](https://www.ncbi.nlm.nih.gov/datasets/taxonomy/335341/) | Influenza_A_H3N2 | ssRNA(-) |
| [GCF_002826225.1](https://www.ncbi.nlm.nih.gov/datasets/taxonomy/1678143/) | Paslahepevirus_balayani | ssRNA(+) |
| [GCF_002889215.1](https://www.ncbi.nlm.nih.gov/datasets/taxonomy/11039/) | Western equine encephalitis virus | ssRNA(+) |
| [GCF_004789475.1](https://www.ncbi.nlm.nih.gov/datasets/genome/GCF_004789475.1/) | Mammerenavirus_choriomeningitidis | ssRNA(+/-) |
| [GCF_009858895.2](https://www.ncbi.nlm.nih.gov/datasets/genome/GCF_009858895.2/) | SARS-CoV-2 | ssRNA(+) |
|  |  |  |
| **Dataset** | **Source** | **Assembly** |
| Transcript | FTP [link](https://ftp.ncbi.nlm.nih.gov/genomes/all/annotation_releases/9606/GCF_000001405.40-RS_2023_10/GCF_000001405.40_GRCh38.p14_translated_cds.faa.gz) | GRCh38.p14 |
| CDS | FTP [link](https://ftp.ncbi.nlm.nih.gov/genomes/all/annotation_releases/9606/GCF_000001405.40-RS_2023_10/GCF_000001405.40_GRCh38.p14_rna.fna.gz) | GRCh38.p14 |

**Supplementary Table S3:** Comparison of average frequency of GG (QP2-7) and GGG (QP3‑7) motifs in human genome assemblies: (A) T2T.CHM13.v2.0 and (B) GRCh.38; Frequency is color-coded from none (red) to high density (green).

| **A** |  | |  |  | **B** |  | |  |
| --- | --- | --- | --- | --- | --- | --- | --- | --- |
|  | **Avg. per Mb** | |  |  |  | **Avg. per Mb** | |  |
| **Chromosome** | **QP2-7** | **QP3-7** | **Ratio** |  | **Chromosome** | **QP2-7** | **QP3-7** | **Ratio** |
| **1** | 1389.4 | 71.4 | 19.5 |  | **1** | 1338.8 | 66.5 | 20.1 |
| **2** | 1200.9 | 57.8 | 20.8 |  | **2** | 1180.5 | 55.7 | 21.2 |
| **3** | 1088.1 | 49.8 | 21.8 |  | **3** | 1088.1 | 47.9 | 22.7 |
| **4** | 913.5 | 42.0 | 21.7 |  | **4** | 918.0 | 40.7 | 22.5 |
| **5** | 1078.7 | 49.8 | 21.6 |  | **5** | 1052.8 | 48.6 | 21.7 |
| **6** | 1091.3 | 51.0 | 21.4 |  | **6** | 1092.0 | 49.5 | 22.1 |
| **7** | 1331.1 | 62.4 | 21.3 |  | **7** | 1298.7 | 61.2 | 21.2 |
| **8** | 1161.6 | 57.6 | 20.2 |  | **8** | 1144.6 | 54.3 | 21.1 |
| **9** | 3100.2 | 61.1 | 50.7 |  | **9** | 1203.5 | 63.6 | 18.9 |
| **10** | 1410.1 | 68.9 | 20.5 |  | **10** | 1369.3 | 65.4 | 20.9 |
| **11** | 1396.6 | 77.7 | 18.0 |  | **11** | 1381.0 | 74.3 | 18.6 |
| **12** | 1306.1 | 64.1 | 20.4 |  | **12** | 1297.6 | 61.2 | 21.2 |
| **13** | 1105.5 | 60.1 | 18.4 |  | **13** | 813.7 | 34.6 | 23.5 |
| **14** | 1487.7 | 66.9 | 22.2 |  | **14** | 1101.4 | 53.2 | 20.7 |
| **15** | 1819.2 | 77.9 | 23.4 |  | **15** | 1209.7 | 57.3 | 21.1 |
| **16** | 1684.5 | 93.5 | 18.0 |  | **16** | 1724.8 | 91.8 | 18.8 |
| **17** | 2182.0 | 119.6 | 18.2 |  | **17** | 2130.2 | 115.6 | 18.4 |
| **18** | 1040.7 | 51.2 | 20.3 |  | **18** | 1027.3 | 46.7 | 22.0 |
| **19** | 2636.4 | 175.4 | 15.0 |  | **19** | 2719.7 | 177.2 | 15.3 |
| **20** | 1810.3 | 96.6 | 18.7 |  | **20** | 1694.9 | 91.4 | 18.6 |
| **21** | 1756.5 | 103.5 | 17.0 |  | **21** | 1120.7 | 59.1 | 19.0 |
| **22** | 2461.1 | 126.8 | 19.4 |  | **22** | 1817.3 | 104.7 | 17.4 |
| **X** | 1052.6 | 55.8 | 18.9 |  | **X** | 1035.4 | 52.9 | 19.6 |
| **Y** | 1578.4 | 29.3 | 53.8 |  | **Y** | 538.1 | 25.2 | 21.3 |
| **Full genome** | 1429.6 | 66.5 | 21.5 |  | **Full genome** | 1238.7 | 61.2 | 20.2 |

**Supplementary Table S4: High resolution RNA quadruplex structures available in the Nucleic Acids Knowledge Base (NAKB** <https://www.nakb.org>) (*)**.** 7 structures correspond to dimers of 2-tetrad quadruplexes. No 2-tetrad monomer was found, except for very complex architectures / long sequences (aptamers) in which multiple additional interactions stabilize the assembly. Structures formed by dimers are highlighted in yellow.

PDB Gene/name Features Length (nt) & sequence for 2-tetrads

1J6S UGAGGU 2+1-tetrads – tetramol 6

1J8G UGGGGU 4 tetrads – tetramol 6

1MDG UGAGGU Octamol 6

1MY9 **2-tetrad (hexads)-dimer** 14 GGAGGUUUUGGAGG

1RHAU UGGGGU 4 tetrads – tetramol 6

2AWE UGGUGU tetrads x 2 – octamer 6

2GRB UGIGGU 4 tetrads – tetramer 6

2M18 TERRA 3-tetrad x 2 – tetramer 10

2KBP TERRA 3-tetrad 12

2LA5 - 3-tetrad (protein) 36

2M18 TERRA 3-tetrad x 2 10

2RQJ Prion aptam **2-tetrad-dimer** 12

2RSK Prion aptam **2-tetrad-dimer** (protein) 12

2RU7 Prion aptam **2-tetrad-dimer (**protein) 12

3IBK TERRA 3-tetrad 12

3MIJ TERRA 3-tetrad (Acridine) 12

4TS0 Spinach complex aptamer (ligand) 44/47

4TS2 Spinach complex aptamer (ligand) 44/47

4KZD Spinach complex aptamer (Fab) 84

4KZE Spinach complex aptamer (Fab) 84

4Q9Q Spinach complex aptamer (Fab) 84

4Q9R Spinach complex aptamer (Fab) 84

4RJ1 UG4U Tetramol. 4 tetrads x 2 6

4RKV UG4U Tetramol. 4 tetrads x 2 6

4RNE UG4U Tetramol. 4 tetrads x 2 6

4TS0 Spinach Complex aptamer (ligand) 44/47

4TS2 Spinach Complex aptamer (ligand) 44/47

4XK0 UG4U Tetramol. 4 tetrads 6

5BJ0 Corn complex aptamer (ligand) 36

5BJP Corn complex aptamer (ligand) 36

5DE5 Fragile X sc1 3 tetrads (protein) 35

5DE8 Fragile X sc1 3 tetrads (protein) 35

5DEA Fragile X sc1 3 tetrads (protein) 35

5OB3 iSpinarch complex aptamer (ligand) 69

5V3F Mango complex aptamer (ligand) 31

6B14 Spinach complex aptamer (protein) 86

6B3K Spinach complex aptamer (protein) 83

6C63 Mango-II complex aptamer (ligand) 36/32

6C64 Mango-II complex aptamer (ligand) 36/32

6C65 Mango-II complex aptamer (ligand) 36/32

6E80 Corn complex aptamer 36

6E81 Corn complex aptamer 36

6E82 Corn complex aptamer 36

6E84 Corn complex aptamer 36

6E8S iMango-III complex aptamer (G2) 38

6E8T Mango-III complex aptamer 35

6E8U Mango-III complex aptamer 37

6C63 Mango-II complex aptamer 36/32

6C64 Mango-II complex aptamer (Biotin) 36/32

6C65 Mango-II complex aptamer (Biotin) 36/35

6GE1 UGGUGGU 2x2 tetrads + U-tetrads 7

6JJH rPRV2L **2-tetrad-dimer** (G4L) 14 GGCUCGGCGGCGGA

6JJI rPRV2L **2-tetrad-dimer** (G4L) 14 GGCUCGGCGGCGGA

6K84 Prion aptam. 4-tetrads ( ?) 25

6PQ7 iMango-III complex aptamer (ligand) 37

6UPO Mango-III complex aptamer (ligand) 38

6V98 Mango-IV complex aptamer (ligand) 28

6V9D Mango-IV complex aptamer (ligand) 28

6XRQ Ea **2-tetrad-dimer** (G4L) 14 GGCUCGGCGGCGGA

7L0Z Spinach var. complex aptamer 69

7MKT r(GU)11 3 tetrads (+U bulges) 23

7OA3 Chili complex aptamer 52

7OAV Chili complex aptamer 52

7OAW Chili complex aptamer 52

7OAX Chili complex aptamer 52

7PS8 U3 3 tetrads 23

7Q48 BCL-2 3 tetrads 22

7Q6L BCL-2 3 tetrads 22

7QA2 BCL-2 3 tetrads 22

7SXP NRAS 3 tetrads 22

8EYU Beetroot complex aptamer 49

8EYV Beetroot complex aptamer 45

8EYW Beetroot complex aptamer 49

8F0N Beetroot complex aptamer 49

8FYH PRC2 (protein) 24

8TNS r(GU)12 3 tetrads (+U bulges) 24

(*) *Result of a search performed on Nov. 29, 2023 using the word “quadruplex” for RNA sequences only. Note that using the NAKB annotations “quadruplex”, “quadruplex-Onquadro annotated” and “G-quadruplex-G4DSSR annotated” gave a lower number of hits. For this reason, the term “quadruplex” was searched anywhere, but required manual curation of structures for which no G-tetrad is observed, or not corresponding to RNA: 1S9L (LNA); 3KIV (riboswitch – no tetrad); 4XBF (ss RNA); 5N94 (poly U); 6DB8 – 6DB9 (aptamer no G4); 8FZA. Screenshot of the search window:*


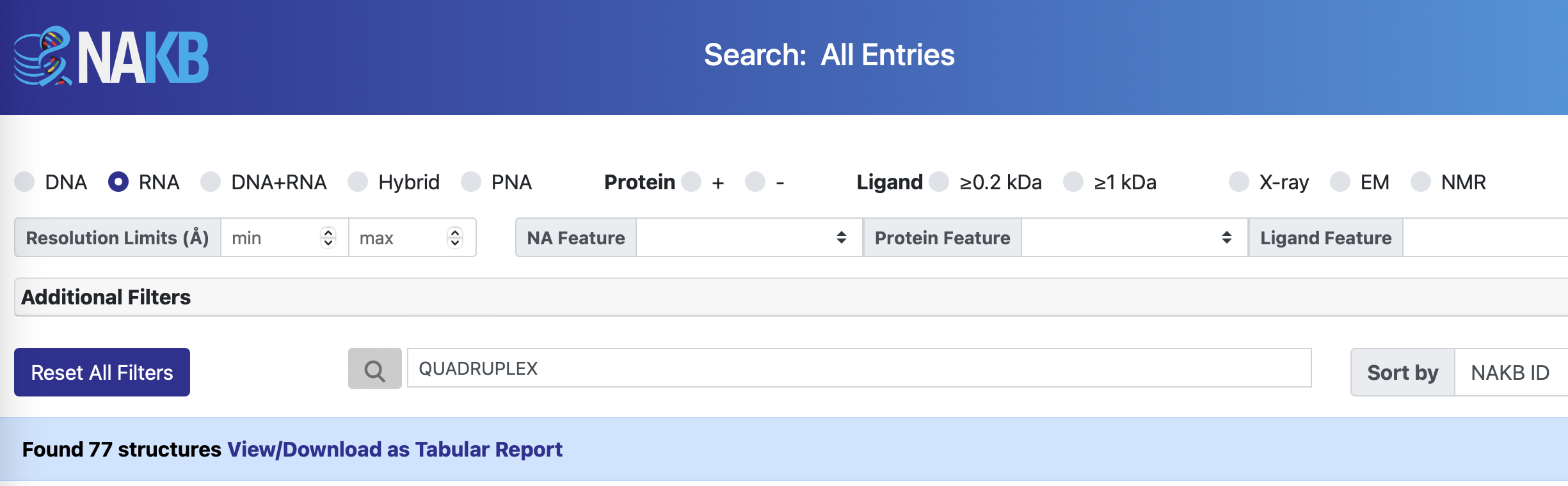


**Fig.** **S1**

*Average frequency of QP2-7/QP3-7 in transcribed regions of hg38 assembly per Mb. 3´UTRs and 5´UTRs analyzed using UCSC RefSeq tracks. CDS - protein coding sequences (merged exons only) and Transcript data (containing UTRs, introns and exons) were obtained from NCBI reposiory.*


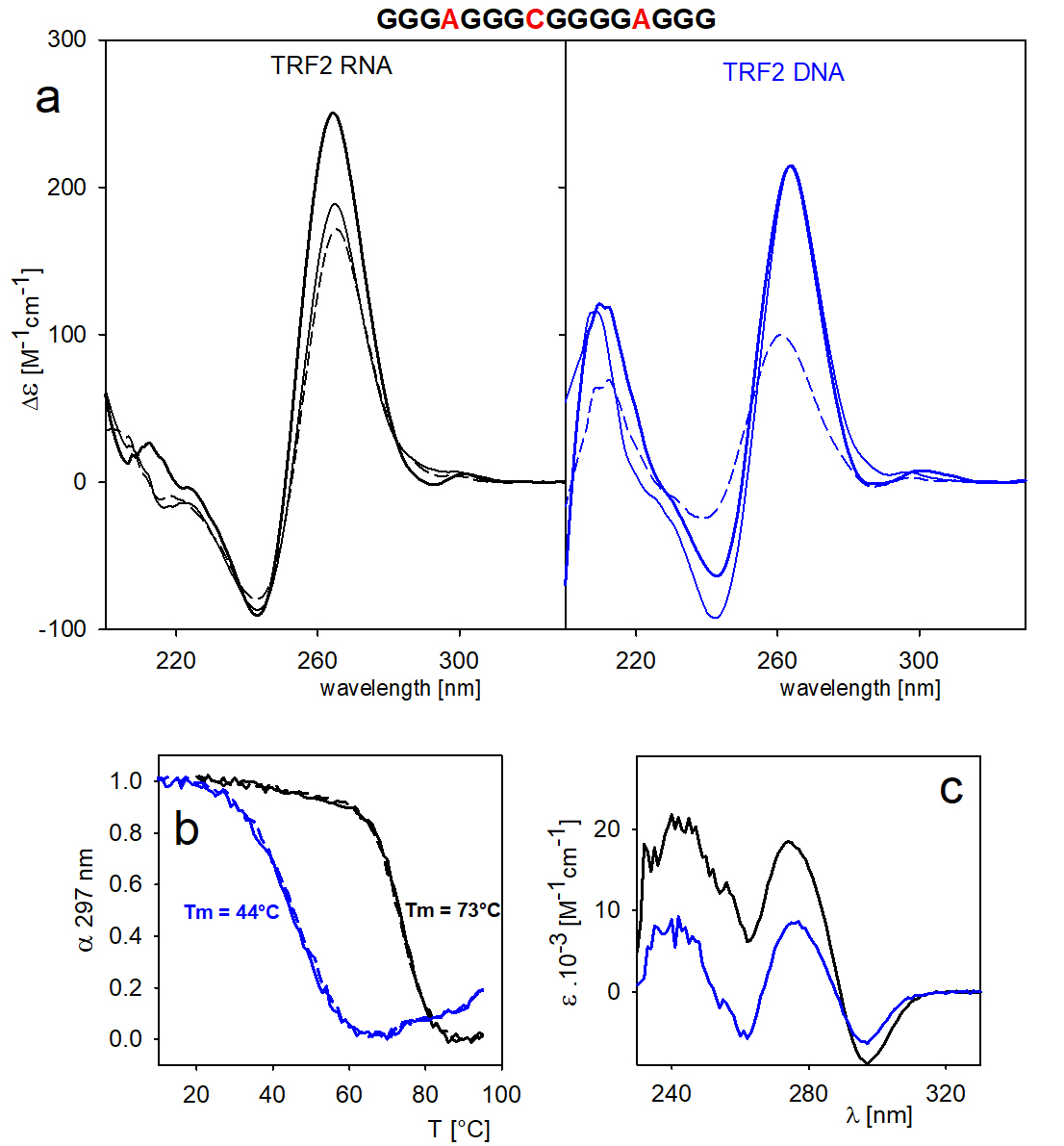


**Fig. S2**

*Comparison of three-tetrad G4 RNA (black) and DNA (blue) sequences: (a) CD spectra at 5 μM concentration measured in (dash lines) 1 mM Na-phosphate, (full lines) 1 mM K-phosphate buffer, (bold lines) 10 mM K-phosphate buffer (15 mM K^+^); (b) normalized thermal melting dependencies at 297 nm in 1 mM K-phosphate (full line) for melting, (dashed line) for renaturation; (c) TDS spectra calculated as the difference of absorption spectra at temperatures with the lowest and highest molar absorption values at 297 nm taken from temperature dependencies (b).*


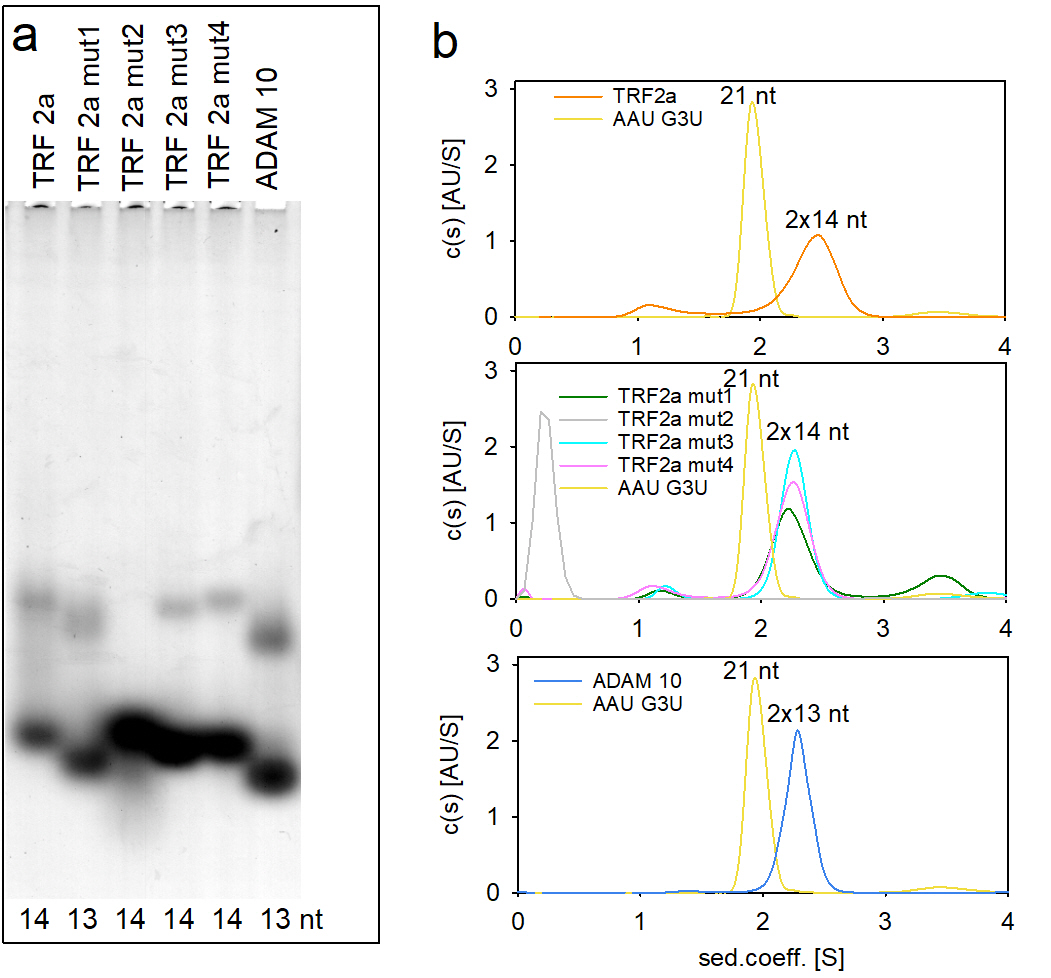


**Fig. S3**

*Comparison of migrations of selected RNA sequences in polyacrylamide gel with sedimentation coefficients determined by analytical ultracentrifugation: (a) 16% PAGE of samples after NMR measurements in 110 mM K^+^ at 23°C performed as described in (Kejnovská, Chemistry – A Eur. J. 2021); (b) AUC measured at 15 μM concentration in 50 mM K^+^: (orange) TRF2a, (green) TRF2a mut1, (grey) TRF2a mut2, (cyan) TRF2a mut3, (pink) TRF2a mut4, (blue) ADAM 10, (yellow) AAU(G_3_U)_3_G3UAA - a marker for 21 bases.*


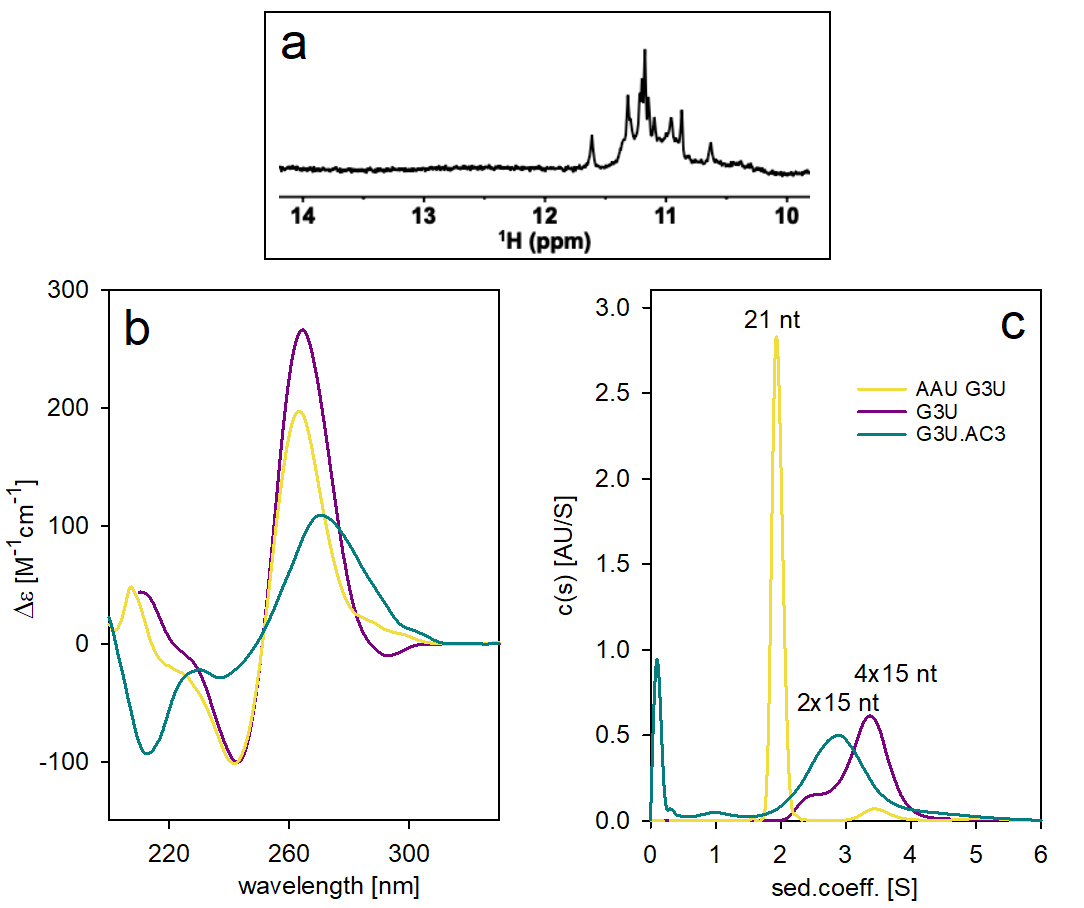


**Fig. S4**

*Comparison of three-tetrad sequences with and without appended nucleotides: (a) NMR spectrum at 90 μM AAU(G_3_U)_3_G_3_UAA in 110 mM K^+^; (b) CD spectra at 5 μM RNA in 110 mM K^+^; (violet) (G_3_U)_3_G_3;_ (yellow) AAU(G_3_U)_3_G_3_UAA and (green) A-form duplex of (G_3_U)_3_G_3_ with a complementary strand C_3_(AC_3_)_3_; (c) AUC measured at 15 μM RNA concentration of the same samples in 50 mM K^+^.*


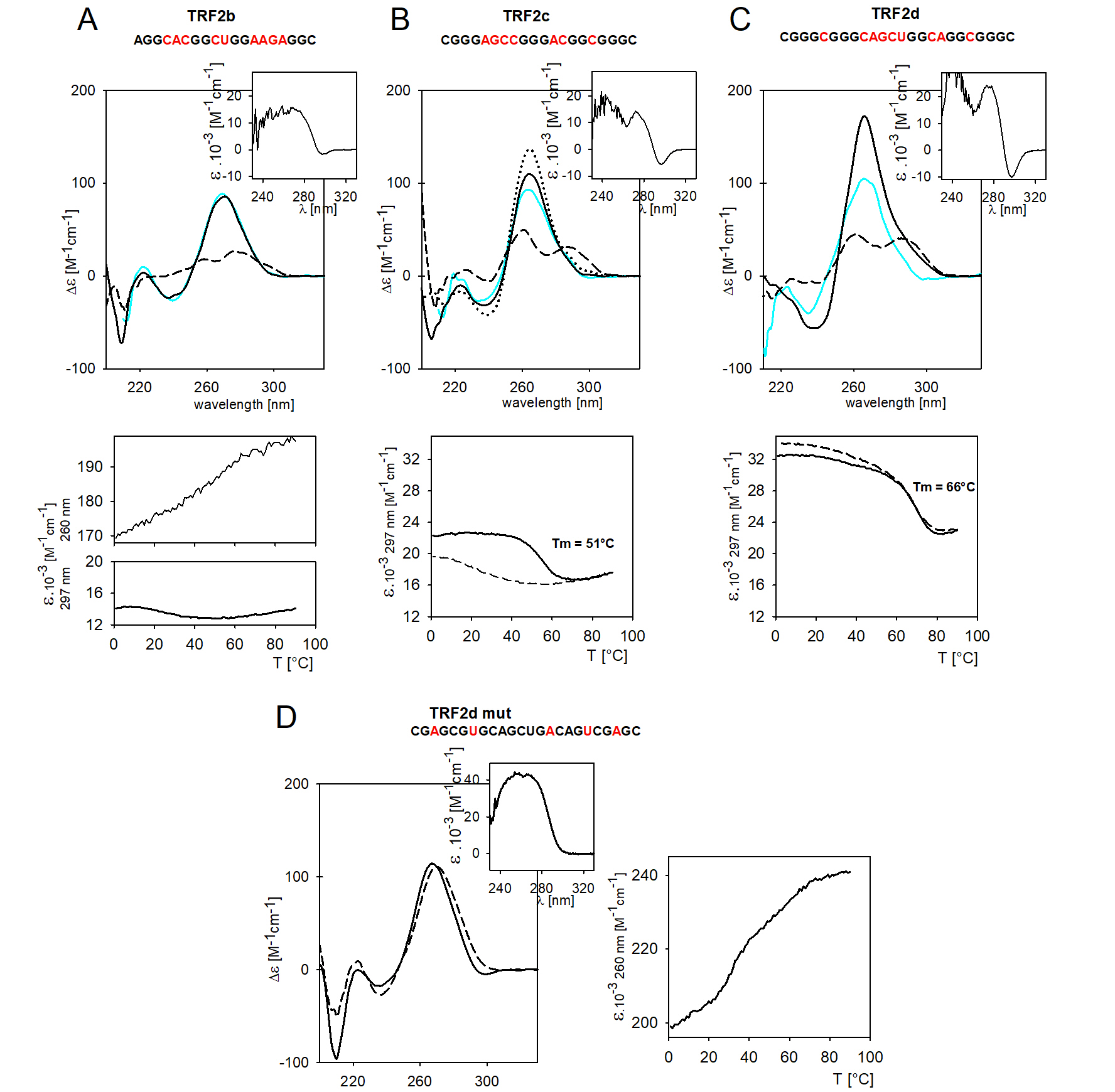


**Fig. S5**

*CD spectra and temperature dependence of (a) TRF2b, (b) TRF2c, (c) TRF2d, and (d) TRF2d mut: Upper row: CD spectra at 8 μM RNA in (black dash) 1 mM Na-phosphate, (black) 110 mM K^+^, (cyan) 110 mM Li^+^ and for TRF2c (black dots) after 24 h in 110 mM K^+^; inserts: TDS in 110 mM K^+^. Bottom rows: Temperature dependence of the same samples in 110 mM K^+^ plotted as molar absorption at 260 nm (for samples that did not form G4; W.C. duplexes are hypochromic), and at 297 nm (for samples forming G4; G4s are hyperchromic at 297 nm); Samples were melted (black line) 24 hours after K^+^ addition; (black dash) renaturation. Melting courses of TRF2b and TRF2d were reversible.*


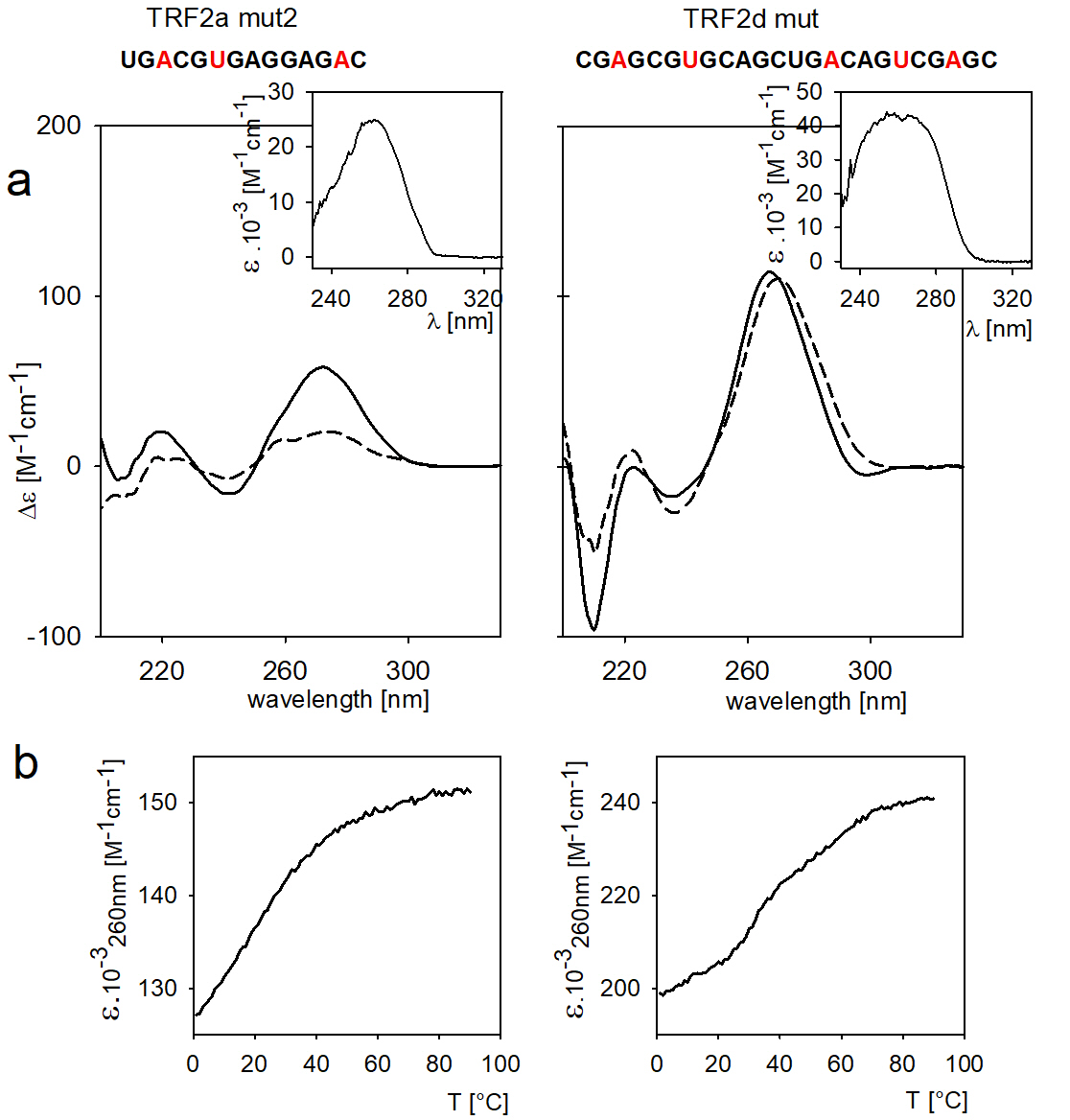


**Fig. S6**

*TRF2a mut2 and TRF2d mut sequences that did not form G4: (a) CD spectra at 8 and 5 μM RNA, respectively, in (black dash) 1 mM Na-phosphate, (black) 110 mM K^+^; inserts: TDS in 110 mM K^+^ measured at 23^o^C. TRF2d mut formed an A-form, while TRF2a mut2 remained unstructured; (b) melting curves monitored at 260 nm: the absorbance of TRF2 mut2 increases monotonously with increasing temperature, TRF2d mut exhibited some indication of cooperativity (A-form duplex or hairpin).*


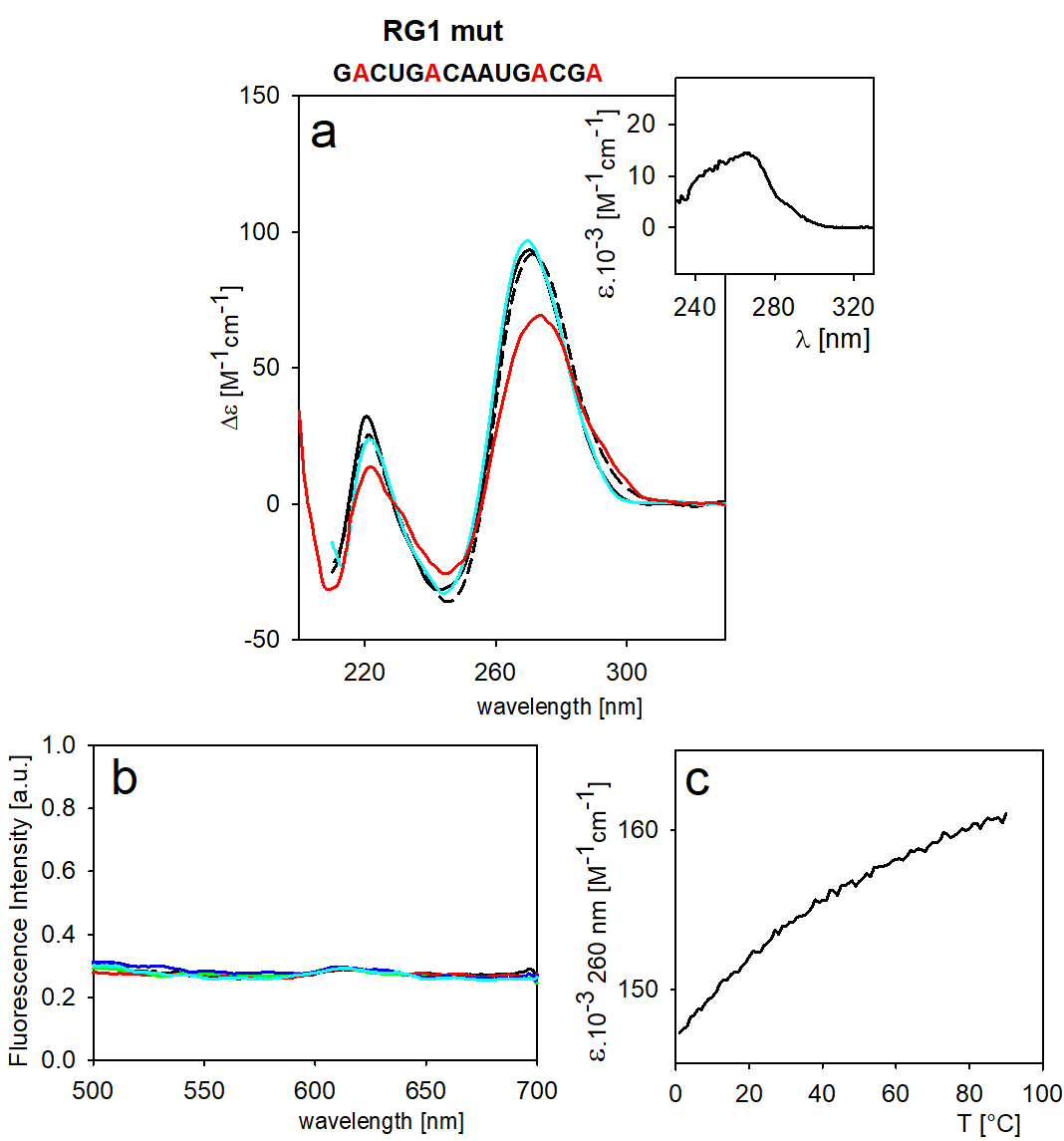


**Fig. S7**

*Analysis of RG1 mut sequence: (a) CD spectra at 5 μM RNA in (black dash) 1 mM Na-phosphate, (black) 110 mM K^+^, and (red) 58 μM RNA in 110 mM K^+^, (cyan) 5 μM RNA in 110 mM Li^+^; insert: TDS taken from melting at 5 μM RNA in 110 mM K^+^; (b) fluorescence measured for increasing RNA concentration (2-8 μM) added to 2 μM NMM; (c) temperature dependence at 260 nm. All results confirmed that this sequence did not form G4.*


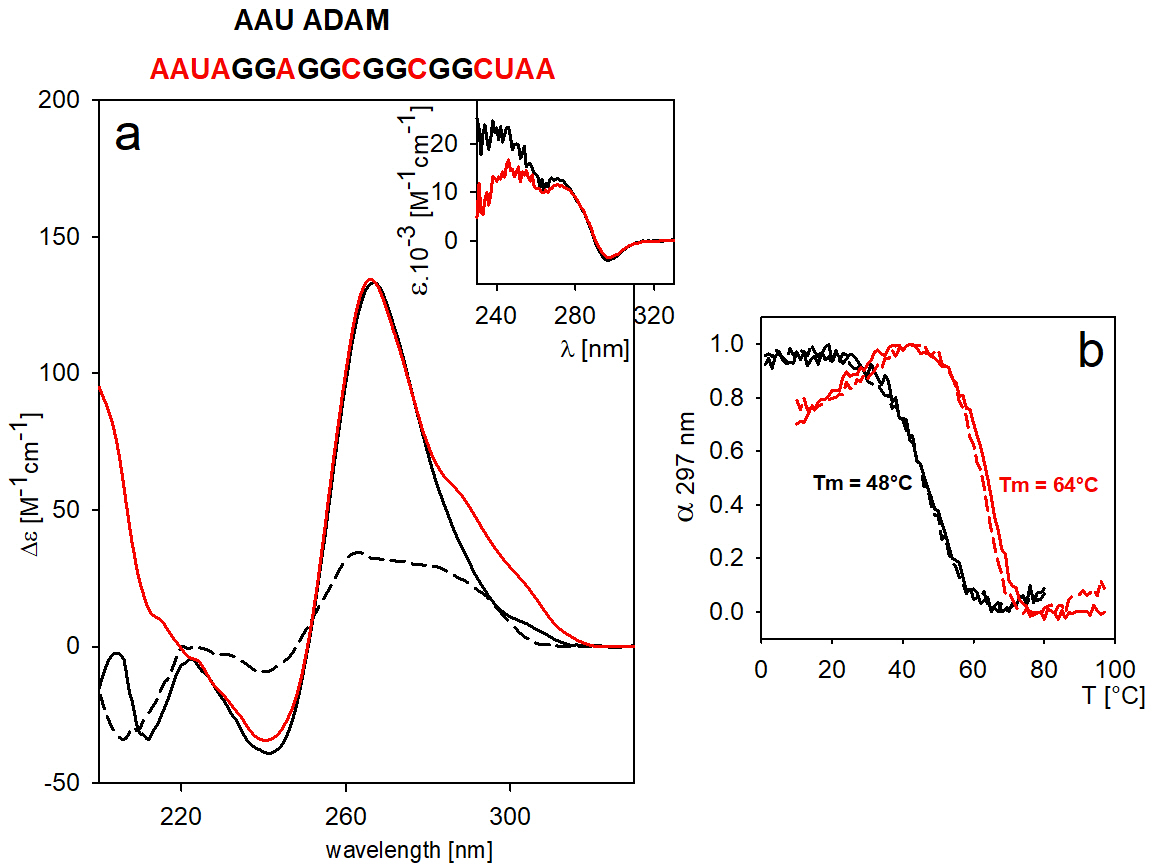


**Fig. S8**

*Analysis of AAU ADAM: (a) CD spectra of 7 μM RNA in (black dash) 1 mM Na-phosphate, (black) 110 mM K^+^, and (red) of 130 μM RNA in 110 mM K^+^, insert: TDS in (black) low and (red) high RNA concentration in 110 mM K^+^; (b) temperature dependence of samples under the same conditions as CD measurements: (solid lines) melting, (dashed) renaturation.*

**b**

**a**

**Fig. S9**

*Comparison of average (a) (GGT)_3_GG (called GGT) and (b) GGTGGT_3_GGTGG (called GGT3) sequence distribution between transcribed and non-transcribed genomic regions of the human genome per 1 Mb.*

*Method:* *NCBI RefSeq Select genomic annotation tracks of 5´UTRs, Introns, Exons, and 3´UTRs for human genome assembly hg.38 were used, downloaded via UCSC Genome Browser. Each hit for sequence motives GGT and GGT3 was count just once. Total counts of sequence motives within transcribed regions were analyzed by BedTools using -intersect function and combined, then subtracted from total number of motives in whole genome referred as non-transcribed genomic regions. Overall distribution of nucleotides was analyzed by Unipro UGENE from FASTA files generated by BedTools -getfasta function from hg.38 assembly. Expected values were calculated according to the total length and representation of each nucleotide in of analyzed region, as follows:*

*Expected = (P_G_)^a^ × (P_T_)^b^ × (R - (k-1))*

*P_G_: probability of guanine occurrence in analyzed DNA region; P_T_: probability of Thymine occurrence in analyzed DNA region; a: number of Guanines in DNA string; b: number of Thymies in DNA string, R: total length of analyzed region (i.e.: human genome, CDS, 3´UTR, 5´UTR, Exons, Introns, -1000) ; k: number of nucleotides in DNA string.*

*Probabilities of G/T occurrence were calculated from composition of all regions corresponding to transcribed regions (5´UTRs, 3´UTRs, Introns, Exons), combined, in respect of their total length and nucleotide distribution. Same approach used for non-transcribed genomic regions. Observed and expected abundancy were calculated as average occurrence per 1 Mb of corresponding regions and compared.*


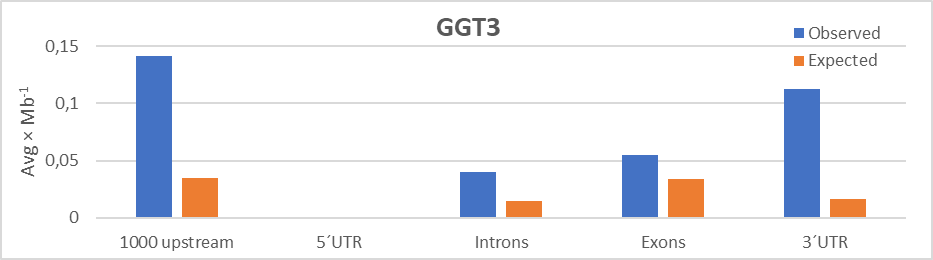


**b**


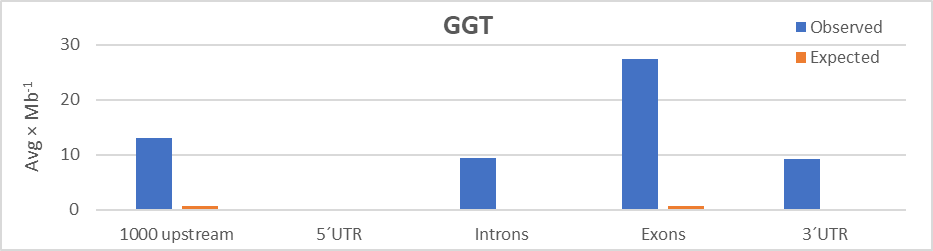


**a1**


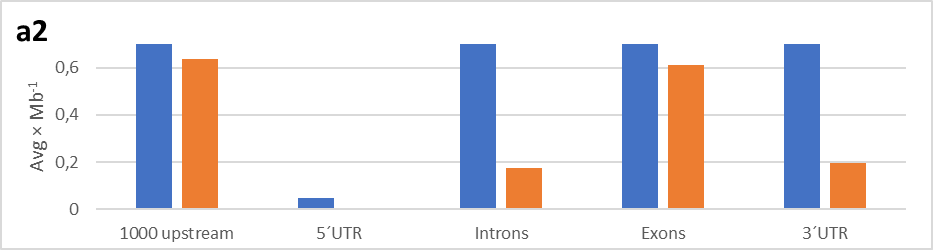


**Fig. S10**

*Comparison of average abundancies of (a1, a2 (zoomed area of a1 with maximum set to 0.7)) GGT and (b) GGT3 sequences in each transcribed region (5´UTR, Introns, Exons, and 3´UTR), and promoter region, referred as 1,000 upstream (of TSS). The number of the GGT/GGT3 sequences observed (blue) is much higher than their number expected (red) based on randomized sequences.*

*Method: The NCBI RefSeq Select genomic annotation tracks of 5´UTRs, Introns, Exons, 3´UTRs and 1000 upstream for human genome assembly hg.38 were downloaded via UCSC Genome Browser. Total counts of sequence motives within each CDS and 1000 upstream of TSS considered as promoter region were analyzed by BedTools using -intersect function. Overall distribution of nucleotides in each region was analyzed by Unipro UGENE from FASTA files generated by BedTools -getfasta function from hg.38 assembly. Expected values were calculated as described previously. Observed and expected abundancy were calculated as average occurrence per 1 Mb of corresponding region and compared.*


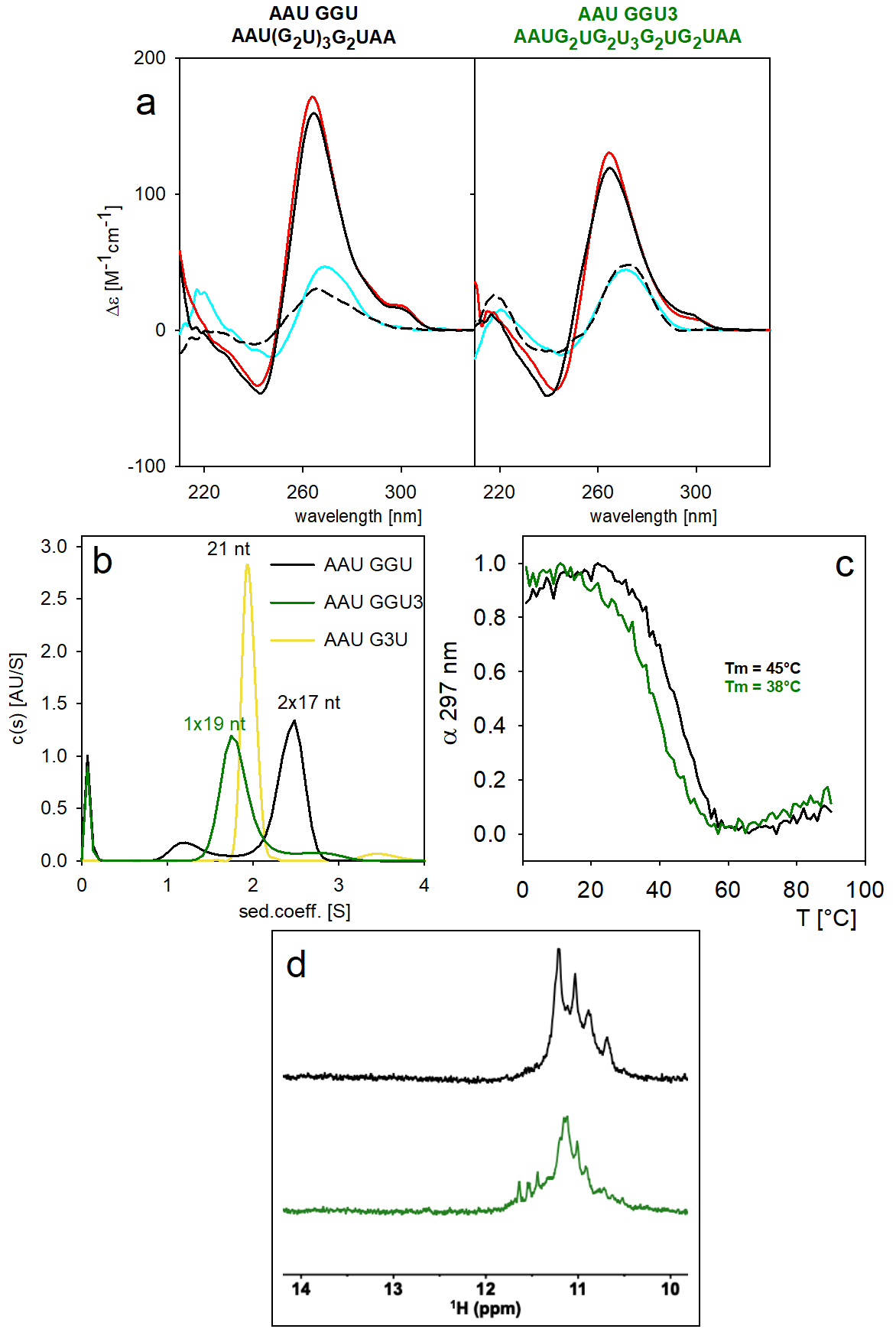


**Fig. S11**

*Comparison of G4s of GG dinucleotides containing sequences (black) AAU(G_2_U)_3_G_2_UAA and (green) AAUG_2_UG_2_U_3_G_2_UG_2_UAA: (a) CD spectra at 8 μM RNA in (black dash) 1 mM Na-phosphate, (black) 110 mM K^+^, (cyan) 110 mM Li^+^, and (red) 140 μM and 130 μM RNA in 110 mM K^+^; (b) AUC of 15 μM samples measured in 50 mM K^+^; (c) normalized temperature dependencies of 8 μM RNA samples in 110 mM K^+^ measured at 297 nm; (d) NMR spectra at 140 μM RNA samples in 110 mM K^+^.*


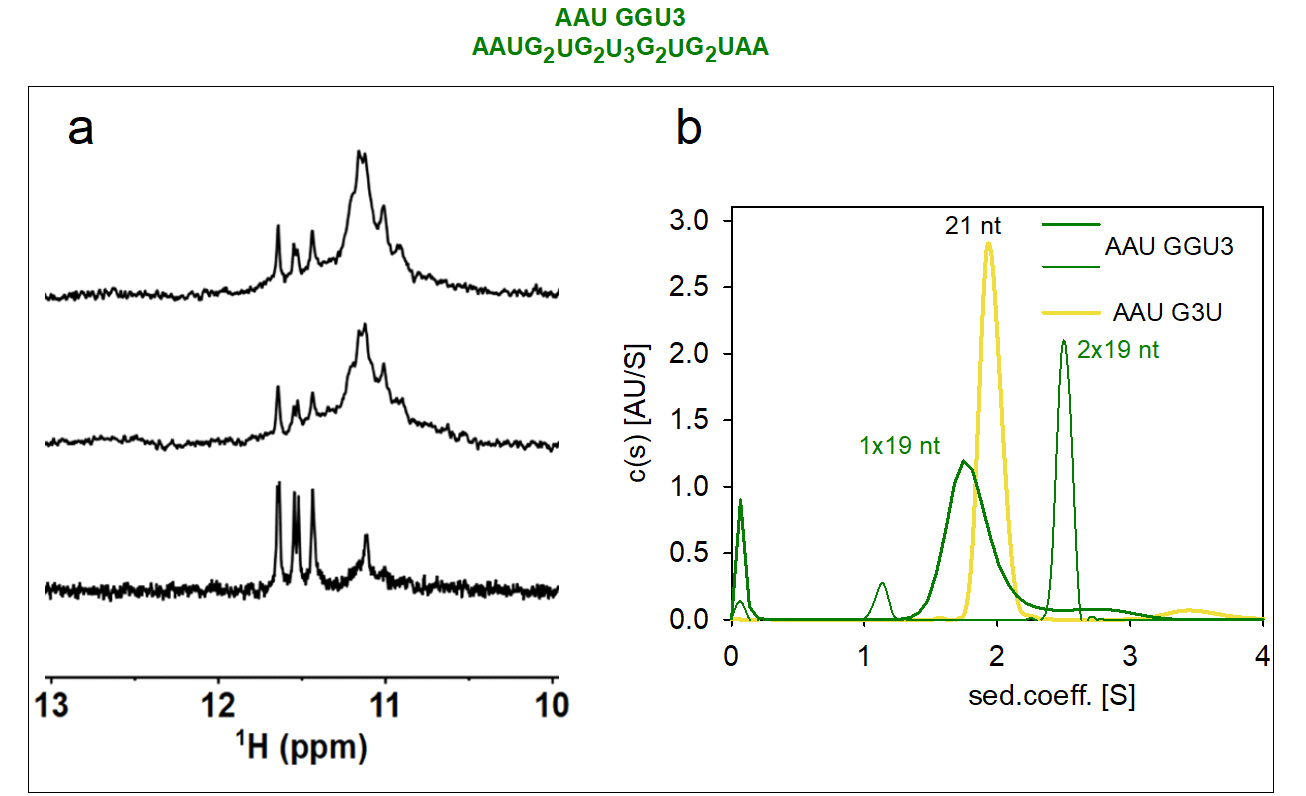


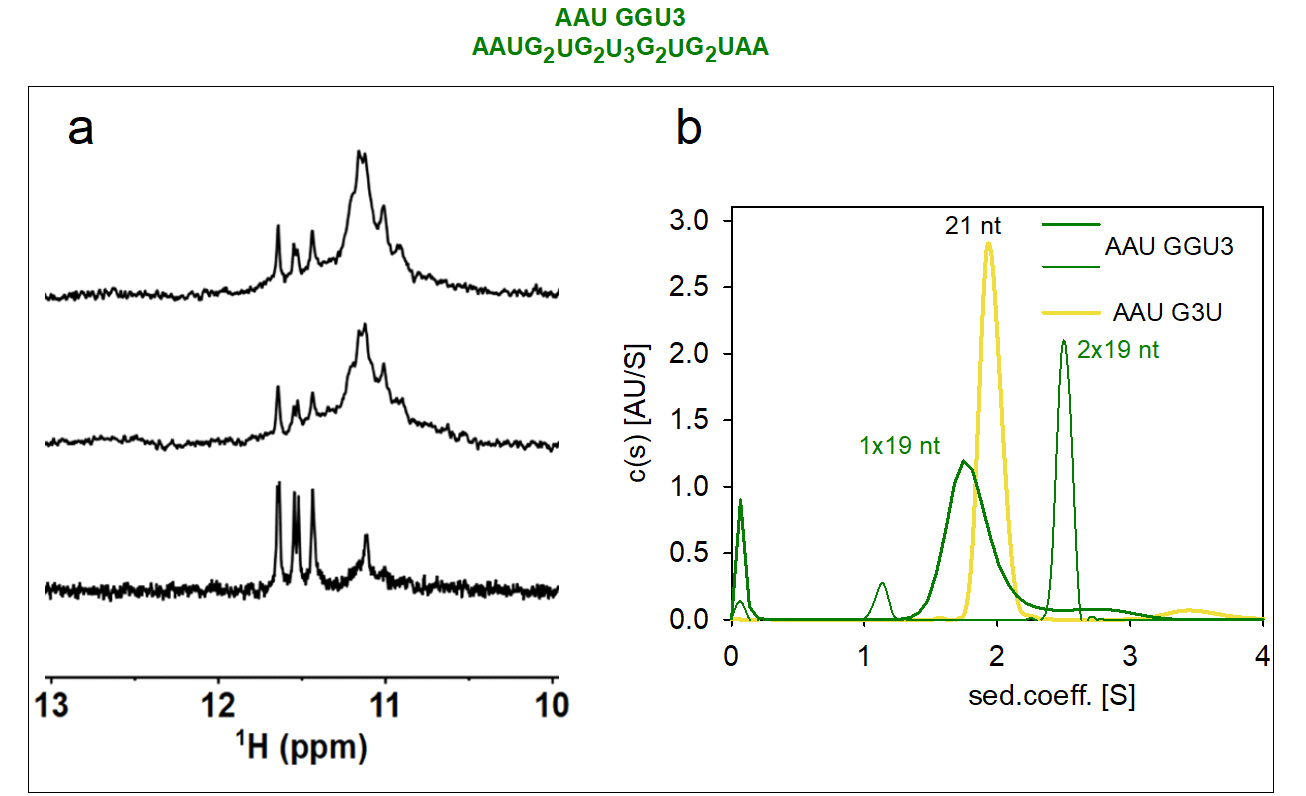


**Fig. S12**

*NMR spectra of AAU GGU3 at 100 μM concentration measured (upper spectrum) immediately, and (middle spectrum) after one day incubation compared to the 30 μM purified AAU GGU3 (bottom spectrum), measured after denaturation and 15 min annealing.*

A


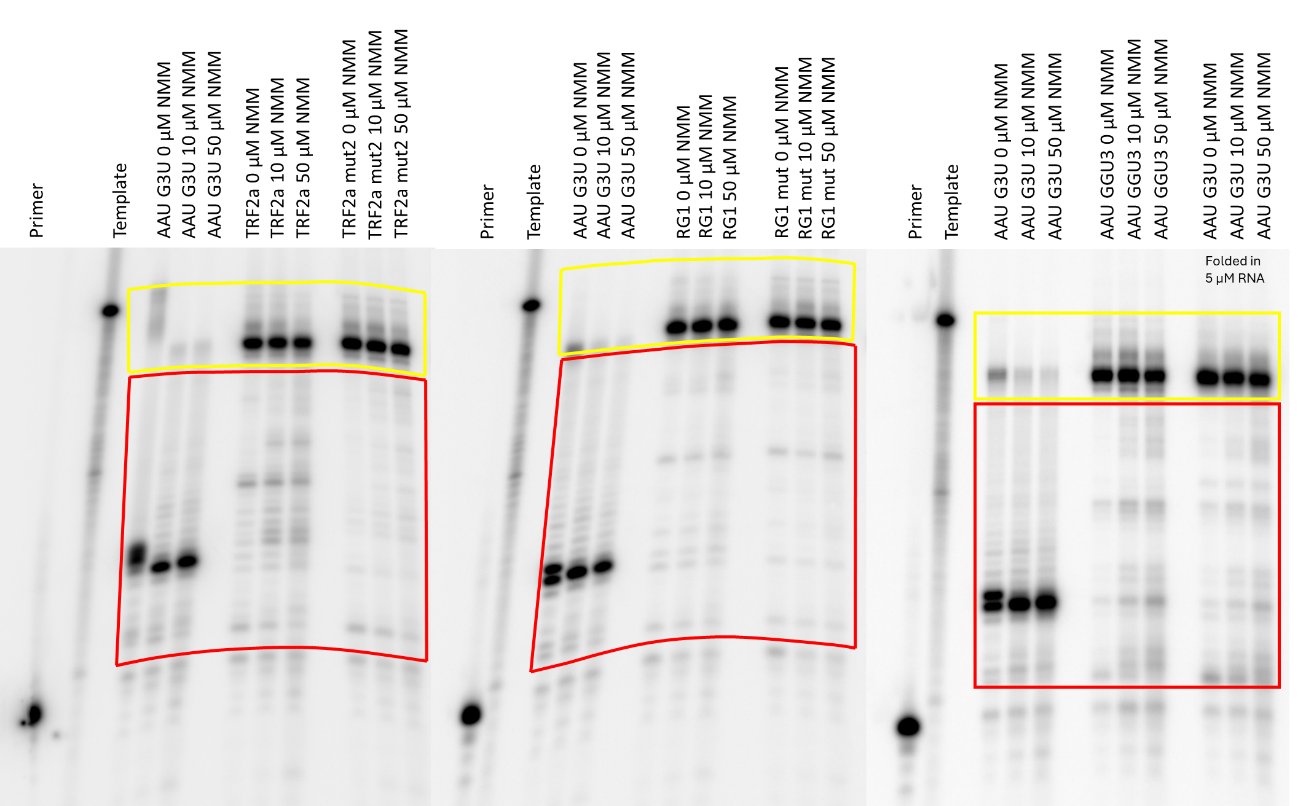


C

B

*
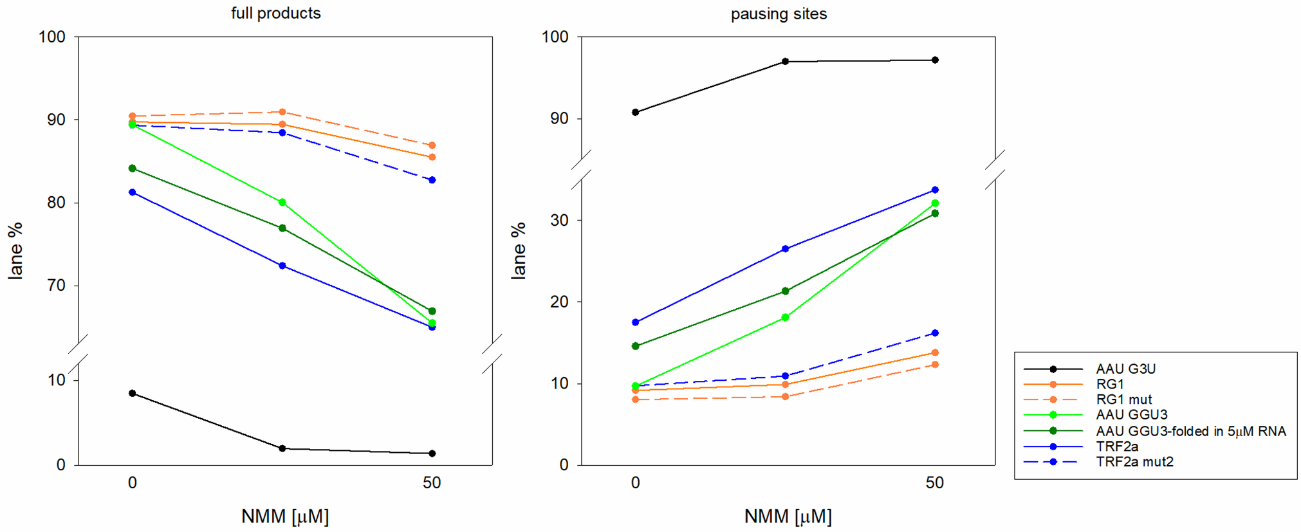
*

**Fig. S13**

*(****A****) Table of oligonucleotides used: The binding sites of primers to the template are shown by underlining, and the RNA product of T7 transcription is indicated in bold. Observed guanine blocks are coloured green, and their mutant variants are marked in red.; (****B****)* *Denaturing PAGE of (yellow frame) full products and (red frame) pausing sites of reverse transcription reaction; (****C****) A graphical comparison of the intensity of selected bands relative to the whole line expressed as a percentage obtained by analysis of denaturing polyacrylamide gels.*

1. *Examples ; all RNA motifs, sequence provided in the 5’ => 3’ direction.* [↑](#footnote-ref-1)
2. *Methods used to identify or characterize G4 formation* [↑](#footnote-ref-2)
